# Proteome profiling of different rat brain regions reveals the modulatory effect of prolonged maternal separation on proteins involved in cell death-related processes

**DOI:** 10.1186/s40659-021-00327-5

**Published:** 2021-02-08

**Authors:** Zdenka Drastichova, Vladimir Rudajev, Gergely Pallag, Jiri Novotny

**Affiliations:** Department of Physiology, Faculty of Science, Charles University, Prague, Czech Republic

**Keywords:** Maternal separation, Brain, Proteomics, Cell deaths, Oxidative stress

## Abstract

**Background:**

Early-life stress in the form of maternal separation can be associated with alterations in offspring neurodevelopment and brain functioning. Here, we aimed to investigate the potential impact of prolonged maternal separation on proteomic profiling of prefrontal cortex, hippocampus and cerebellum of juvenile and young adult rats. A special attention was devoted to proteins involved in the process of cell death and redox state maintenance.

**Methods:**

Long-Evans pups were separated from their mothers for 3 h daily over the first 3 weeks of life (during days 2–21 of age). Brain tissue samples collected from juvenile (22-day-old) and young adult (90-day-old) rats were used for label-free quantitative (LFQ) proteomic analysis. In parallel, selected oxidative stress markers and apoptosis-related proteins were assessed biochemically and by Western blot, respectively.

**Results:**

In total, 5526 proteins were detected in our proteomic analysis of rat brain tissue. Approximately one tenth of them (586 proteins) represented those involved in cell death processes or regulation of oxidative stress balance. Prolonged maternal separation caused changes in less than half of these proteins (271). The observed alterations in protein expression levels were age-, sex- and brain region-dependent. Interestingly, the proteins detected by mass spectrometry that are known to be involved in the maintenance of redox state were not markedly altered. Accordingly, we did not observe any significant differences between selected oxidative stress markers, such as the levels of hydrogen peroxide, reduced glutathione, protein carbonylation and lipid peroxidation in brain samples from rats that underwent maternal separation and from the corresponding controls. On the other hand, a number of changes were found in cell death-associated proteins, mainly in those involved in the apoptotic and autophagic pathways. However, there were no detectable alterations in the levels of cleaved products of caspases or Bcl-2 family members. Taken together, these data indicate that the apoptotic and autophagic cell death pathways were not activated by maternal separation either in adolescent or young adult rats.

**Conclusion:**

Prolonged maternal separation can distinctly modulate expression profiles of proteins associated with cell death pathways in prefrontal cortex, hippocampus and cerebellum of juvenile rats and the consequences of early-life stress may last into adulthood and likely participate in variations in stress reactivity.

**Supplementary Information:**

The online version contains supplementary material available at 10.1186/s40659-021-00327-5.

## Background

The maternal separation (MS) paradigm, in which pups are separated from their dams for long periods of time (1–6 h per day) during first two or three weeks after delivery, is often used as a model of early-life stress (ELS). Exposure to ELS belongs among the risk factors for developmental programming of adverse outcomes in adulthood [1]. An increasing number of studies published in the past two decades have been attempting to delineate the biological mechanisms of ELS [2]. A wide range of consequences of exposure to ELS was identified, involving morphological, neuroendocrine, behavioral, metabolic and epigenetic alterations in the offspring’s development. These changes can underlie the increased susceptibility to infectious, metabolic, cardiovascular and psychiatric diseases in later life [1]. Neuropsychiatric diseases include depressive, anxiety and autism spectrum disorders as well as schizophrenia [3]. The large individual variability and disease susceptibility arise from the interaction between biological (e.g., sex, age of assessment, predisposing genetic polymorphisms in genes and epigenetic signature in genes) and environmental factors (e.g., nature of stressors, timing and duration of exposure during development, severity and cumulative exposure effects, maternal care and health, nutrition) [3, 4].

ELS has a great impact on brain development and neuroplasticity because the brain undergoes extensive developmental processes and organizational changes during the perinatal period. Insults encountered during this period as well as other biological and environmental factors may affect early-life programming of the nervous system, thereby having the potential to influence brain functioning in adulthood [1, 5, 6]. A theory, known as the cumulative stress hypothesis, pronounces that individuals are more likely to suffer from disease as life adversity accumulates. On the other hand, the match/mismatch hypothesis suggests that early-life adversity may prepare an organism for exposure to similar (“matching”) adversity later in life and produce a predictive adaptive response to optimize responses to future stressor, while a disease is developing if a mismatch occurs between the early programming environment and the later adult environment [6, 7]. The latter hypothesis considers that ELS can have either beneficial or adverse consequences depending on the context of specific biological and environmental factors.

The nervous system undergoes developmental processes (e.g., neurogenesis, synaptogenesis, dendritic and axonal arborization, and cell death) during the embryonic period as well as in adolescence. Neurogenesis, including proliferation and differentiation of neural stem cells, migration, survival, maturation and integration of newborn neurons, proceeds in adulthood and declines with age. In rats, neurogenesis in cortex is largely completed at birth, while a wave of secondary neurogenesis occurs in cerebellum and hippocampus during the first three postnatal weeks. More than eighty percent of neurogenesis in the dentate gyrus region of the hippocampus occurs after birth with a peak during the first three weeks and continues through adulthood [8, 9]. In rodents, migration of neurons in cortex is diminished at birth but it continues until PND 20 in cerebellum and proceeds through both adolescence and adulthood in hippocampus [10, 11].

The spontaneous reduction in the number of NSCs may be regulated by programmed cell death, reduced mitotic potential and terminal differentiation [5, 12, 13]. Developmentally programmed cell death in the nervous system occurs naturally at various stages of embryonic and postnatal development, while pathological cell death executed in neurodegenerative disorders is referred to as regulated cell death. A number of different forms of cell death (e.g., apoptosis, autophagy and necrosis) have been described and although their mechanisms and morphologies differ they may overlap at signaling level. Apoptosis as a counterpart of mitosis and cell proliferation is critical during developmental processes [14–16]. Neurons are highly susceptible to programmed cell death because more than 50% of newly generated neurons are eliminated in certain brain regions during development. However, this susceptibility decreases during development, suggesting that the expression and activity of components of the apoptotic machinery are temporally modulated [17, 18]. Apoptotic cell death in cortex and hippocampus reaches the highest level at PND 1. Consequently, the apoptotic process is strongly suppressed during the first 3 postnatal weeks and apoptosis rate reaches a plateau at PND 90 [19]). The cerebellar apoptosis reaches its peak at PND 10, followed by a small increase at PND 21 and a low, basal plateau during adult life [19]. While neurogenesis and neuron migration in cortex proceeds differently than in hippocampus and cerebellum, developmental cell death occurs similarly in cortex and hippocampus, but in a slightly different way in cerebellum. Although postmitotic cells, including differentiated neurons, are resistant to apoptosis, programmed cell death is part of adult neurogenesis in which neural stem cells differentiate into multiple cell types (e.g. neurons, astrocytes, and oligodendrocytes), and integration of young neurons into existing neural circuits [13, 20]. Developmental programmed as well as regulated cell death depend on the input/output neuronal activity (e.g., synaptogenesis, neurotransmission), mitochondrial function, calcium buffering capacity and redox signaling and perturbations in these processes may contribute to the pathogenesis of neurological and psychiatric disorders [13, 21, 22].

Oxidative stress is considered to be one of the main factors contributing to the development of neurological and psychiatric disorders [23, 24]. Oxidative stress represents disturbed redox homeostasis due to the imbalance between production of reactive oxygen species (ROS) and function of antioxidant defense systems. The excessive generation of ROS, which are not sufficiently eliminated by antioxidant system, causes a damage of biomolecules such as lipids, proteins and nucleic acids resulting in necrosis and apoptotic cell death. The brain is susceptible to oxidative damage because it consumes a large amount of oxygen and contains high amount of lipids prone to peroxidation [24, 25]. Only few studies examined the possible relationship between maternal separation and the development of oxidative stress and the existing results are undefined. The effect of maternal separation on antioxidant enzyme activities and lipid peroxidation in different rat brain regions were found to be age-dependent [26], sex-dependent [27, 28] as well as brain region-dependent [27, 29]. The hippocampus seems to be less prone to alterations in oxidative state induced by maternal separation than the cortex [27, 29, 30]. Moreover, maternal deprivation, when pups are separated once for 24 h, induces more alterations in oxidative state [26, 29] than maternal separation (3–6 h per day repeatedly during the first three weeks of postnatal life) [27, 28, 30].

In this study, we investigated which biological processes are affected in response to prolonged maternal separation. We were eager to explore the effect of maternal separation on cell death proteome in order to reveal whether maternal separation provokes cell death and may be beneficial or harmful in connection with altered developmental processes or the development of neurodegenerative disorders. It is known that the developing brain is markedly susceptible to oxidative stress and neuronal apoptosis [31]. Therefore, we evaluated the potential role of oxidative state as a possible mechanism involved in regulating cell death. The development of oxidative stress as a consequence of maternal separation is indefinite and may be strongly dependent on experimental conditions. While the consequences of oxidative stress induced by maternal deprivation might be harmful in later life stages, mild stress evoked by maternal separation could possibly prepare adaptive responses to stressful stimuli encountered in adult life. Here, we focused on determining protein expression profiles in three different brain regions (cortex, hippocampus and cerebellum) in juvenile rats as well as male and female adult rats. Cerebral cortex and hippocampus are key regulators of the HPA axis, which structure and function is known to be altered by ELS [32]. The HPA axis dysregulation was observed in some neurodegenerative disorders [33]. The consequences of maternal separation on cerebellum have been only rarely studied. Lupien et al. [34] reported that prolonged early maternal separation in rats increased the density of CRH (corticotropin releasing hormone) binding sites in cerebellum and that such an increase in CRH-binding sites in the brain can have negative effects. We suspect that maternal separation may have a considerable impact on structure and function of the cerebellum. Therefore we have included this brain region to our proteomic analyses.

## Materials and methods

### Animals and housing

Male and female Long-Evans rats (approximately 8 weeks of age) were purchased from Velaz, Ltd., Prague, Czech Republic. Upon arrival, rats were singly housed in standard plastic cages containing wood chip bedding. They were maintained at normal ambient temperature (22 ± 1 °C) under a stable light–dark cycle (12 h light and 12 h darkness), and were allowed free access to food and water. All procedures were performed according to national and institutional guidelines for the care and use of animals in laboratory research. The protocols were approved by the Ministry of Education, Youth and Sports the Czech Republic (licence no. MSMT-43092/2014-5).

### Maternal separation

**Fig. 1:**
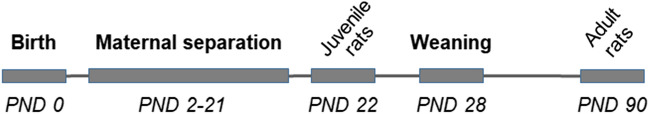
Experimental outline. The experimental timeline is shown. From PND 2 to PND 21, pups from randomly selected litters were subjected to unpredicted maternal separation. They were individually isolated from their mothers for 3 h a day. Roughly half of the control and maternally separated animals (“juvenile rats”) were sacrificed at PND 22 and the other half of the rats were weaned at P28 and housed in same-sex littermate groups until PND 90 (“adult rats”)

We chose prolonged maternal separation (3 h per day for three weeks) as a transitional paradigm between harmful pro-apoptotic effects of maternal deprivation [35] and possible beneficial anti-apoptotic effects of brief maternal separation [36]. Ten dams and litters were subjected to separation for 3 h per day from postnatal day 2 (PND 2) to PND 21 and other ten dams and litters served as controls. All litters were treated in the same way. They were kept together (except for the maternal separation paradigm) until use for analyses. Litter size was between 9 and 18 animals with random distribution of the male/female ratio. The timing of separation was unpredictable, but was always during the light phase. The experimental setup is summarized in Fig. 1. During maternal separation (MS), a whole litter of pups were always transported to another room, to prevent olfactory and visual communication with their mother. The pups were placed separately in individual small plastic boxes on a Sanitas SHK28 heating pad (Hans Dinslage GmbH, Baden-Württemberg, Germany). The temperature of the heating pad was set at 35 ± 1 °C during PND 2–11, and 28 ± 1 °C during PND 12–21. After the 3 h separation, the pups were returned to the maternity cages. The control animals were left undisturbed with their mothers except during the weekly cage cleaning, reflecting a small amount of handling. On PND 22, randomly selected groups of juvenile maternally separated and control animals were sacrificed and their brains were dissected with a scalpel. The brain parts (prefrontal cortex, hippocampus and cerebellum) were immediately frozen in liquid nitrogen and stored at − 80 °C. The remaining maternally separated and control pups were allowed to stay with their dams until weaning (PND 28), then separated from the dam, sexed and housed in groups of a maximum of 6 same-sex individuals until PND 90. Comparative proteomic analysis was performed on cerebrocortical, hippocampal and cerebellar tissues from 22- to 90-day-old rats. PND 22 was chosen as the point of development at which maternal separation was discontinued. The collection of brain tissue samples at this time point allowed evaluating the acute effects of maternal separation. At this age, the brain reaches 90–95% of adult weight, the mature aerobic cerebral metabolism is attained, and cerebellar growth in thickness is completed [9]. On the other hand, PND 90 was chosen in order to detect the potential long-term impact of maternal separation in adult rats as it is known that ELS is a risk factor for the development of neurodegenerative disorders in adulthood [1].

### Brain tissue homogenization

Brain tissue samples from 30 juvenile and 20 adult rats in each group were used to generate sufficient material for testing. Pooled samples of prefrontal cortex and the whole hippocampus and cerebellum were cut into small pieces and homogenized in 9 volumes of ice-cold PBS supplemented with protease inhibitor cocktail Complete (Roche) in a Potter–Elvehjem Teflon–glass homogenizer with the pestle rotating at 1200 r.p.m. using 10 up-and-down strokes. The homogenate was centrifuged at 600×*g* for 10 min (4 °C) to remove unbroken cells and larger debris. The postnuclear supernatant was used to prepare samples for proteomic mass spectrometry analysis or snap frozen in liquid nitrogen and stored at − 80 °C until use for further experiments. Protein concentration was determined by a standard BCA assay.

### nLC-MS^2^ proteomic procedures and analyses

The postnuclear supernatant was mixed 1:1 with 100 mM triethylammonium bicarbonate buffer containing 2% (w/v) sodium deoxycholate and sonicated using three 10-s bursts of a Bandelin UW 2070 sonicator (at 50% amplitude). Sonicated lysates were cleared by centrifugation (14,000×*g*, 10 min, 4 °C). Protein concentration of the supernatant was adjusted to 1 mg/ml, samples were aliquoted into new tubes and stored − 80 °C. Twenty µg of protein per sample was used for mass spectrometry sample preparation. Cysteins were reduced with 10 mM final concentration of tris(2-carboxyethyl)phosphine and blocked with 40 mM final concentarion of chloracetamide (30-min incubation at 60 °C). Samples were digested with trypsine (trypsin/protein ration 1/30) at 37 °C overnight. After digestion samples were acidified with trifluoroacetic acid to 1% final concentration. Sodium deoxycholate was removed by extraction with ethyl acetate and peptides were desalted using in-house made stage tips packed with C18 disks (Empore) according to Rappsilber et al. [37].

Nano reversed phase columns (EASY-Spray column, 50 cm × 75 µm ID, PepMap C18, 2 µm particles, 100 Å pore size) were used for LC/MS analysis. Mobile phase buffer A was composed of water and 0.1% formic acid. Mobile phase B was composed of acetonitrile and 0.1% formic acid. Samples were loaded onto the trap column (C18 PepMap100, 5 μm particle size, 300 μm × 5 mm, Thermo Scientific) for 4 min at 18 μl/min loading buffer was composed of water, 2% acetonitrile and 0.1% trifluoroacetic acid. Peptides were eluted with Mobile phase B gradient from 4 to 35% B in 120 min. Eluting peptide cations were converted to gas-phase ions by electrospray ionization and analyzed on a Thermo Orbitrap Fusion (Q-OT- qIT, Thermo Scientific). Survey scans of peptide precursors from 350 to 1400 m/z were performed in orbitrap at 120 K resolution (at 200 m/z). Tandem MS was performed by isolation at 1,5 Th with the quadrupole, HCD fragmentation with normalized collision energy of 30, and rapid scan MS analysis in the ion trap. The MS2 ion count target was set to 104 and the max injection time was 35 ms [38].

All data were analyzed and quantified with the MaxQuant software (version 1.6.3.4). The false discovery rate (FDR) was set to 1% for both proteins and peptides and we specified a minimum peptide length of seven amino acids. The Andromeda search engine was used for the MS/MS spectra search against the Rattus Norvegicus database (containing roughly 29 000 proteins). Enzyme specificity was set as C-terminal to Arg and Lys, also allowing cleavage at proline bonds and a maximum of two missed cleavages. Carbamidomethylation of cysteine was selected as fixed modification and N-terminal protein acetylation and methionine oxidation as variable modifications. The “match between runs” feature of MaxQuant was used to transfer identifications to other LC–MS/MS runs based on their masses and retention time (maximum deviation 0.7 min) and this was also used in quantification experiments. Quantifications were performed with the label-free algorithm in MaxQuant [39]. Data analysis was performed using the Perseus software (version 1.6.1.3) [40]. The proteins whose levels differed by at least twofold between any two groups were considered differentially expressed.

Gene Ontology (GO) enrichment analysis of the whole set of differentially expressed proteins was performed using the GOrilla web interface (http://cbl-gorilla.cs.technion.ac.il); the p-value threshold was set to 10^–5^ for biological processes, and the remaining settings were the defaults in the tool. To our knowledge, the GOrilla tool is suitable to deal with large sets of gene expression data (in the order of thousands). Therefore, GO enrichment analysis of differentially expressed proteins for each experimental group, containing a smaller set of items (in the order of hundreds), was carried out using the ShinyGO v0.61 tool (bioinformatics.sdstate.edu/go). For each experimental group, the cutoff of p-value (FDR) was set to 0.05 and the top fifty most significantly enriched GO terms for biological processes were summarized in the form of hierarchical cluster tree dendrograms complemented with p-values.

### Western blot analysis

Postnuclear supernatants from brain homogenates were mixed in a 1:3 ratio with 4X Laemmli loading buffer containing β-mercaptoethanol and the proteins (20 μg/lane) were resolved by SDS-PAGE under denaturing conditions, followed by Western blotting. The nitrocellulose membrane, following protein transfer, was rinsed briefly with double-distilled water and blocked with 5% nonfat dry milk. After incubation with specific primary antibodies, blots were washed and incubated with appropriate secondary antibodies coupled to HRP. Bound antibodies were revealed by enhanced chemiluminescence (ECL) and exposure to X-ray film [41]. The images generated were quantitatively analyzed for the protein levels with the use of ImageJ software.

### Assessment of oxidative stress markers

Redox balance in selected brain regions was assessed by estimating the level of glutathione (GSH), malondialdehyde (MDA), lipid hydroxyperoxides (LOOH) and protein carbonyls.

Determination of GSH was based on the reaction of Ellman’s reagent (DNTB) with sulfhydryl groups, which yields a yellow colour measurable at 412 nm [42]. The absorbance of the reduced chromogen is directly proportional to GSH concentration. Briefly, brain tissue homogenates were mixed in a 1:1 ratio with 10% (w/v) trichloroacetic acid (TCA) and centrifuged at 5000 rpm for 10 min at 4 °C (Hettich Mikro 200R centrifuge). The supernatant (100 μl) was transferred to a new tube, followed by the addition of 400 μl 0.3 M disodium hydrogen phosphate buffer, 50 μl H_2_O and 80 μl 1 μM DTNB (freshly prepared). After 10 min of dark incubation, absorbance was measured with a microplate reader Synergy HT (Biotek) and reduced glutathione was used as a standard.

The MDA levels in brain homogenates were determined using a TBARS method [43]. Briefly, the reaction mixture was prepared by mixing 100 μl of tissue homogenate, 200 μl 10% TCA and 300 μl 0.67% 2-thiobarbituric acid (TBA). The reaction mixture was heated at 90 °C for 30 min. After 5-min cooling on ice, samples were centrifuged at 10,000 rpm. Supernatant absorbance was measured at 535 nm. 1,1,3,3-Tetramethoxyopropane was used as a standard for constructing the calibration curve to calculate the concentration of MDA.

Lipid hydroperoxides (LOOH) were estimated by the ferrous oxidation-xylenol orange (FOX) assay [44]. The FOX reagent consisted of 20% methanol, 25 mM H_2_SO_4_, 4 mM butyrylated hydroxytoluene, 250 μM ferrous ammonium sulfate and 100 μM xylenol orange. The assay was initiated by the addition of 100 µl of brain homogenate to 900 µl of the FOX reagent. The mixture was incubated for 30 min at room temperature and absorbance determined at 560 nm. LOOH were quantified by reference to a calibration curve obtained with H_2_O_2_ standard solutions.

Protein carbonyl content was determined by the derivatization of protein carbonyl groups with 2,4-dinitrophenylhydrazine (DNPH) leading to the formation of stable chromophoric dinitrophenylhydrazones [45]. Briefly, the brain homogenate (200 μl) was precipitated with an equal volume of 1% TCA and centrifuged at 5000 rpm for 10 min at 4 °C. The pellet was resuspended with 10 mM DNPH and 2 N HCl. After washing with 1:1 ethanol-ethylacetate, the final pellet was dissolved in 6 M guanidine. Carbonyl content was determined from the absorbance at 370 nm using a molar absorption coefficient of 22,308 M^−1^ cm^−1^ and related to protein concentration.

### Determination of glutathione peroxidase activity

Glutathione peroxidase (EC 1.6.4.2) is one of the most important enzymes for detoxification of peroxides in living cells. Glutathione peroxidase (GPx) activity was measured at 25° C by the method of Flohé and Gunzler [46] with some modifications. Briefly, 100 μl of brain postnuclear supernatant (100 μg) was added to a reaction mixture (100 μl) consinting of 50 mM sodium phosphate buffer (pH 7.0), 2 mM sodium azide, 2 mM GSH, 200 μg/mL NADPH and 0.05 U of glutathione reductase, and incubated at 25° C for 10 min. After addition of 5 μl of H_2_O_2_ (0.027%) to each well, the samples were measured at 340 nm for 300 s in a spectrophotometer. Glutathione peroxidase activity was standardized against protein concentrations and expressed as nmol/min/mg protein.

### Statistics

Statistical analyses were performed using Graph Pad Prism 7.0 (Graph Pad Software, Inc., San Diego, CA, USA). All data are presented as averages ± S.E.M. and were analyzed using one-way ANOVA followed by Tukey's multiple comparison test. Differences between averages were considered statistically significant when *p* < 0.05.

## Results and discussion

### Comparison of the protein profiles of cerebral cortex, hippocampus and cerebellum

**Fig. 2:**
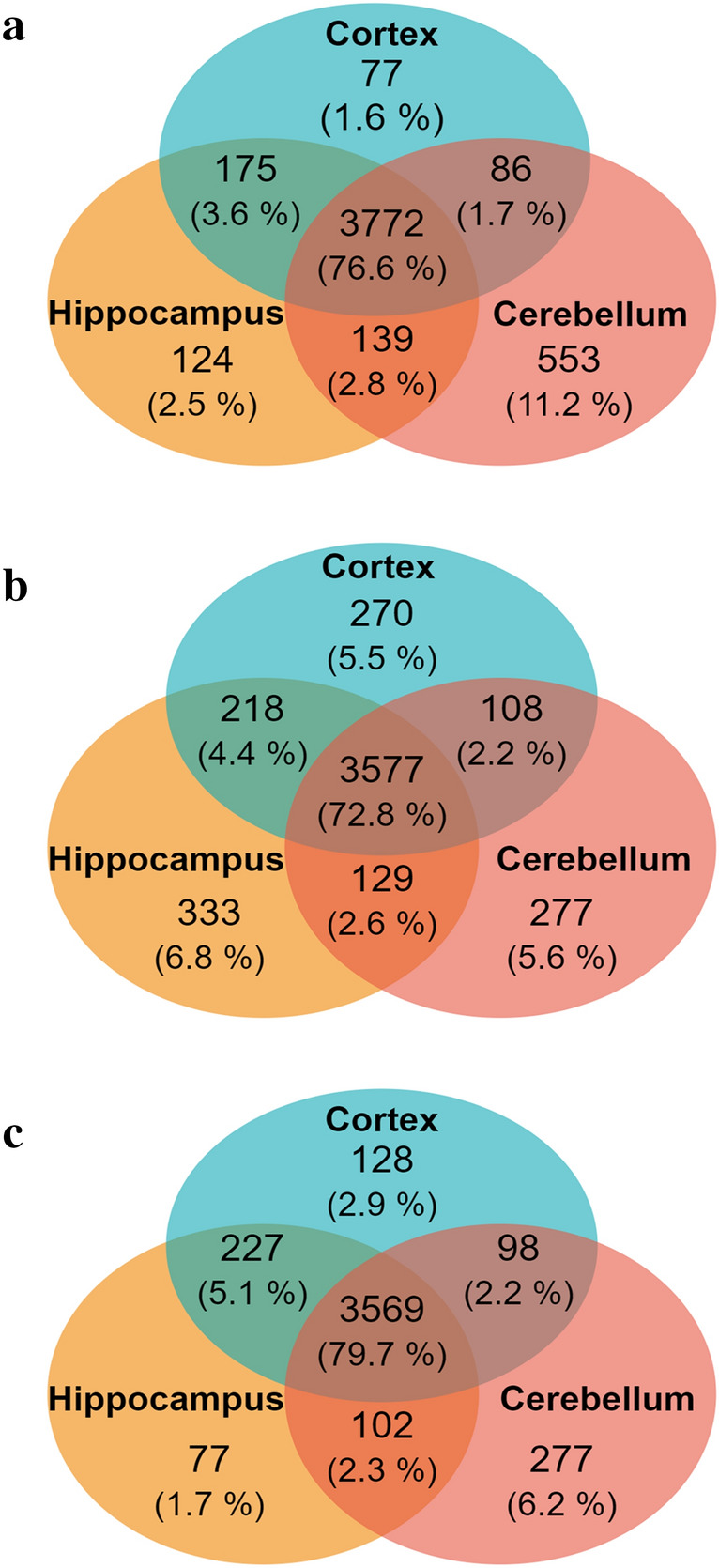
Distribution of proteins identified in the three selected brain regions. All proteins determined by label-free mass spectrometry were divided according to their localization in prefrontal cortex, hippocampus and cerebellum of juvenile (**a**), adult male (**b**) ad adult female (**c**) rats. Proportions (both raw numbers and percentages) of shared and unique proteins in the three brain regions are visualized using Venn diagrams

In order to evaluate the presumed differences in brain regional proteomes of juvenile and young adult rats, bioinformatics analysis of data acquired by bottom-up label-free LC–MS proteomics approach was conducted using the MaxQuant and Perseus software platforms. A total of 5526 proteins were identified. The proteins were detected at least in one sample of control or maternally separated rats. There were 4926, 4912 and 4478 proteins found in all cerebrocortical, hippocampal and cerebellar proteomes of juvenile, male and female rats, respectively, indicating that the female proteome consists of a smaller number of proteins detectable by mass spectrometry than the male one. Figure 2 provides a graphical depiction of the proportions of proteins determined in each rat group (juvenile, male or female rats) relative to their distribution in three selected brain regions. The largest part of the proteome in all three groups occurred simultaneously in all three brain regions (cortex, hippocampus and cerebellum). There were a higher number of overlapping proteins in these three brain regions in females (79.7%, Fig. 2c) than in males (72.8%, Fig. 2b). By comparing the proportions of proteins present simultaneously in two brain regions, we observed that there were markedly greater numbers of common proteins in cortex and hippocampus than in cortex and cerebellum or the hippocampus and cerebellum (Fig. 2a–c). Analysis of proteins detected in only one of the three brain regions showed that the number of unique proteins in the cerebellum was markedly higher than that in the cortex and hippocampus of juvenile and adult female rats but not male rats (Fig. 2a). Interestingly, the proportions of proteins detected in only one brain region of adult male rats were similar (Fig. 2b). These data suggest that the rat cerebellar proteome differs from the proteome of rat cortex and hippocampus.

**Fig. 3:**
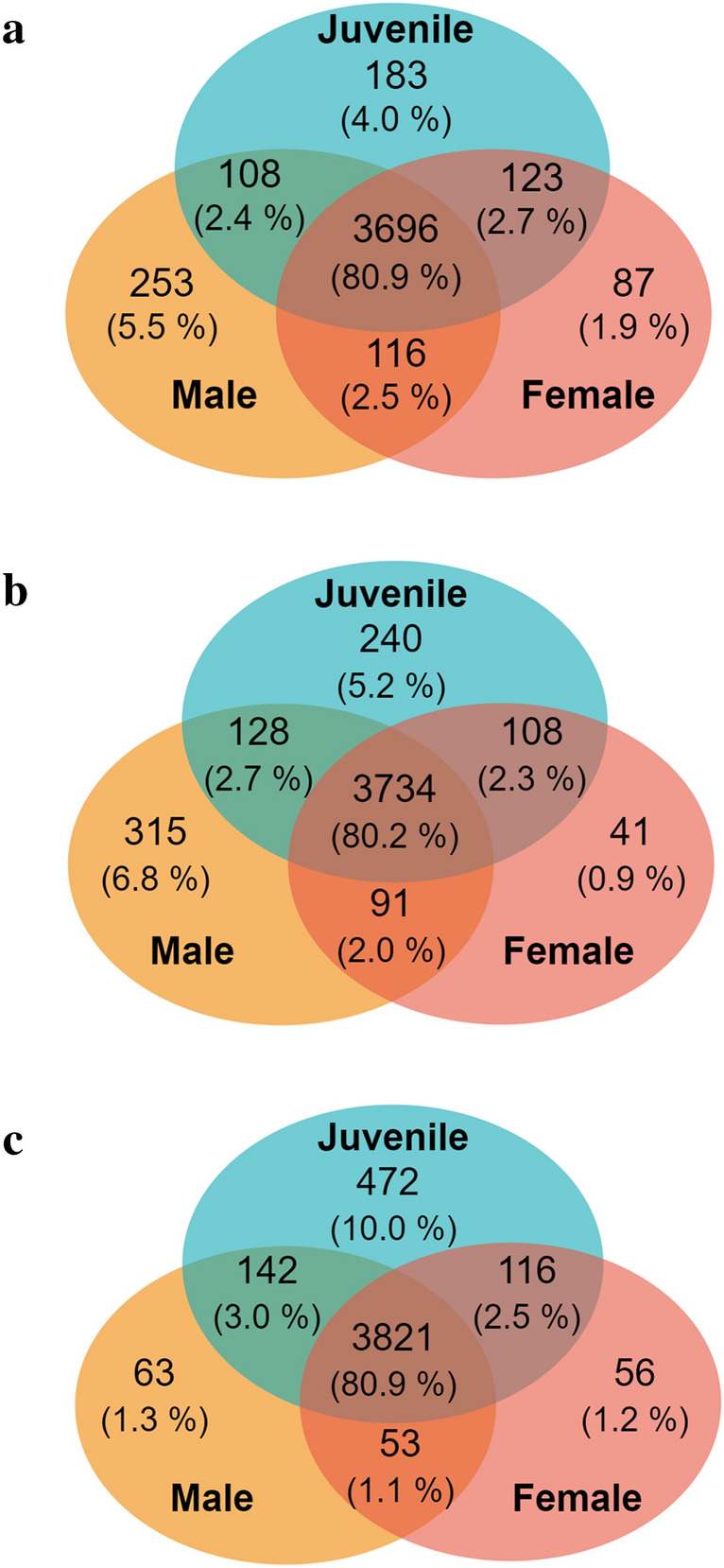
Distribution of proteins identified in juvenile, male and female rats. All proteins determined by label-free mass spectrometry were divided according to their localization in prefrontal cortex (**a**), hippocampus (**b**) and cerebellum (**c**) of juvenile, adult male and adult female rats. Proportions (both raw numbers and percentages) of shared and unique proteins in different age/sex groups are visualized using Venn diagrams

Ranking of all proteins according to their localization showed 4566 proteins in cortex, 4657 proteins in hippocampus and 4723 proteins in cerebellum. Next, these subsets of proteins were divided according to their occurrence in juvenile, male or female rats (Fig. 3). In each brain region, 80% of proteins were concurrently detected in juvenile, male and female rats. The proportion of proteins present simultaneously in two age/sex groups (juvenile/male, juvenile/female and male/female) ranged between 2–3%, except for proteins present in both male and female cerebellum, which represented only about 1.1% of the total number of identified proteins (Fig. 3c). In cortex and hippocampus, there were far fewer proteins found only in female rats, compared with the number of proteins found only in male or juvenile rats. In cerebellum, the proportion of proteins found only in juvenile rats was notably higher than that in cortex and hippocampus. Besides that there were relatively small numbers of proteins found only in male rats or female rats as compared with those identified in juvenile rats. Interestingly, the most unique protein expression profile was seen in cerebellum of juvenile rats.

### GO enrichment analysis of proteins altered by maternal separation

**Fig. 4:**
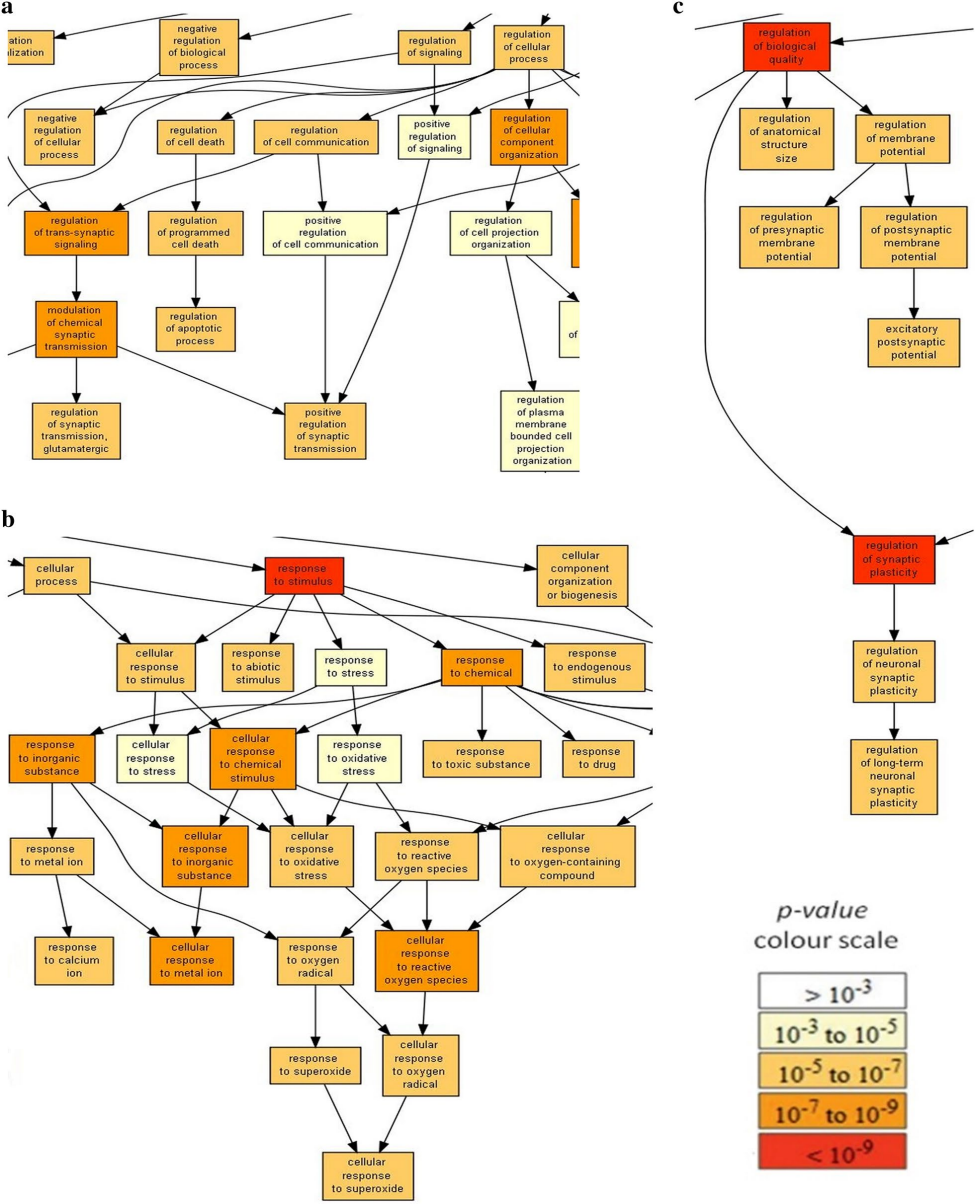
Summary of gene ontology enrichment analysis of differently expressed proteins after prolonged maternal separation. GO enrichment analysis was conducted using the Gorilla tool (http://cbl-gorilla.cs.technion.ac.il/) on 2735 proteins altered at least in one of nine pairwise comparisons. The GO terms in biological processes with p-value < 10^–5^ were analyzed. The color indicates the degree of enrichment, from red (very significantly enriched) to white (not enriched). The results confirmed the enrichment of the GO terms that are related to the regulation of cell death and apoptotic processes, as well as to the regulation of glutamatergic synaptic transmission (**a**), response to stimulus, response to oxidative stress and reactive oxygen species, response to calcium ion (**b**), biological quality and synaptic plasticity (**c**). A detailed overview of GO enrichment analysis is presented in Additional file 1: Fig. S1 and Additional file 2: Table S1

From a total of 5526 proteins, the levels of 2735 proteins were altered by maternal separation at least in one of nine pairwise comparisons. There were 1643 altered proteins identified with a null q-value. Gene Ontology (GO) enrichment analysis was conducted on 2735 differently expressed proteins using the GOrilla tool (http://cbl-gorilla.cs.technion.ac.il). The system recognized 1717 genes out of 2735 gene terms and 1634 of these genes were associated with a GO term. The GO terms with p-value < 10^–5^ for biological processes are presented in Additional file 1: Fig. S1 and Additional file 2: Table S1. The analysis confirmed the enrichment of proteins involved in the regulation of cell death and apoptotic processes, glutamatergic synaptic transmission (Fig. 4a), response to stimulus, response to oxidative stress and reactive oxygen species, response to calcium ion (Fig. proteomic procedures), as well as biological quality and synaptic plasticity (Fig. 4c). The most enriched GO terms were related to the regulation of biological quality (p-value = 1.02E−10 and FDR q-value = 8.09E−7), response to stimulus (p-value = 1.35E−10 and FDR q-value = 5.37E−7) and synaptic plasticity (p-value = 9.93E−10 and FDR q-value = 2.63E−6).

GO enrichment analysis of individual experimental groups was performed using the ShinyGO v0.61 tool (bioinformatics.sdstate.edu/go) and the results of hierarchical clustering are presented as dendrograms in Additional file 3: Fig. S2. The following enriched GO terms were frequently detected in several experimental groups: organelle and component organization and biogenesis, protein complex assembly, transport and localization, catabolic processes, RNA processing and splicing, protein- or peptidyl-amino acid modification, and chromosome organization and segregation. The enriched GO terms related to programmed cell death and autophagy of peroxisome were found in male cortex (p-value = 9.8E−3) and female cortex (p-value = 4.8E−3), respectively. The enriched GO terms related to response to nitrogen compounds were found in cerebellum of adult rats of both sexes (p-value = 2.1E−6 in males and p-value = 2.4E−3 in females). Alterations of synaptic proteins were found in juvenile hippocampus (p-values from 1.4E−2 to 7.6E−3), juvenile cerebellum (p-value = 2.1E−2), male cerebellum (p-values from 3.6E−5 to 1.9E−6) and female cortex (p-values from 4.7E−3 to 1.5E−3). Changes in the glutamate receptor signaling pathway were found in juvenile cerebellum (p-values from 3.6E−2 to 1.7E−2) and male cerebellum (p-value = 3.6E−9). The enriched GO terms related to neurogenesis, neuron differentiation and development were found in male cerebellum (p-values from 3.9E−5 to 1.1E−7).

Protein classification was based on functional annotations using the GO knowledgebase for biological processes, molecular function and cellular component categories. A total of 586 proteins were found to be involved in cell death, apoptosis and/or regulation of redox homeostasis according to GO biological processes or to have antioxidant activity according to GO molecular function. These proteins were sorted based on their q-values, arranged in alphabetical order and subsequently classified according to their role in cellular physiological processes (Additional file 4: Table S2). Out of these, the function of 393 proteins is related to cell death and apoptotic processes, while only 125 proteins play a role in oxidative stress, redox homeostasis, glutathione metabolism or antioxidant defense. Sixty-eight proteins are involved in both biological processes.

### Proteomic profiling of rat brain after maternal separation

The impact of prolonged maternal separation on selected protein profiling was studied in samples of cortex, hippocampus and cerebellum of juvenile and adult male or female rats using label-free quantification (LFQ). Samples from rats subjected to maternal separation were always compared with age-matched controls (denoted S/C). Next, we also assessed the differences between protein levels in brain tissue samples from control and maternally separated males and females.

Pairwise comparison of proteins listed in Additional file 4: Table S2 revealed 271 qualitative or quantitative changes in protein expression (Additional file 5: Table S3). Qualitative changes were defined as those when pairwise comparison revealed certain proteins only in one sample. No less that twofold differences between groups were considered as quantitative alterations. One hundred and eighty proteins were identified with a null q-value and score higher than 4. The qualitative alterations were denoted by the letter C (proteins detected exclusively in control samples) or S (proteins detected exclusively in samples from maternally separated rats). Sex differences were denoted by the letter M (proteins detected exclusively in male samples) or F (proteins detected exclusively in female samples).

**Fig. 5:**
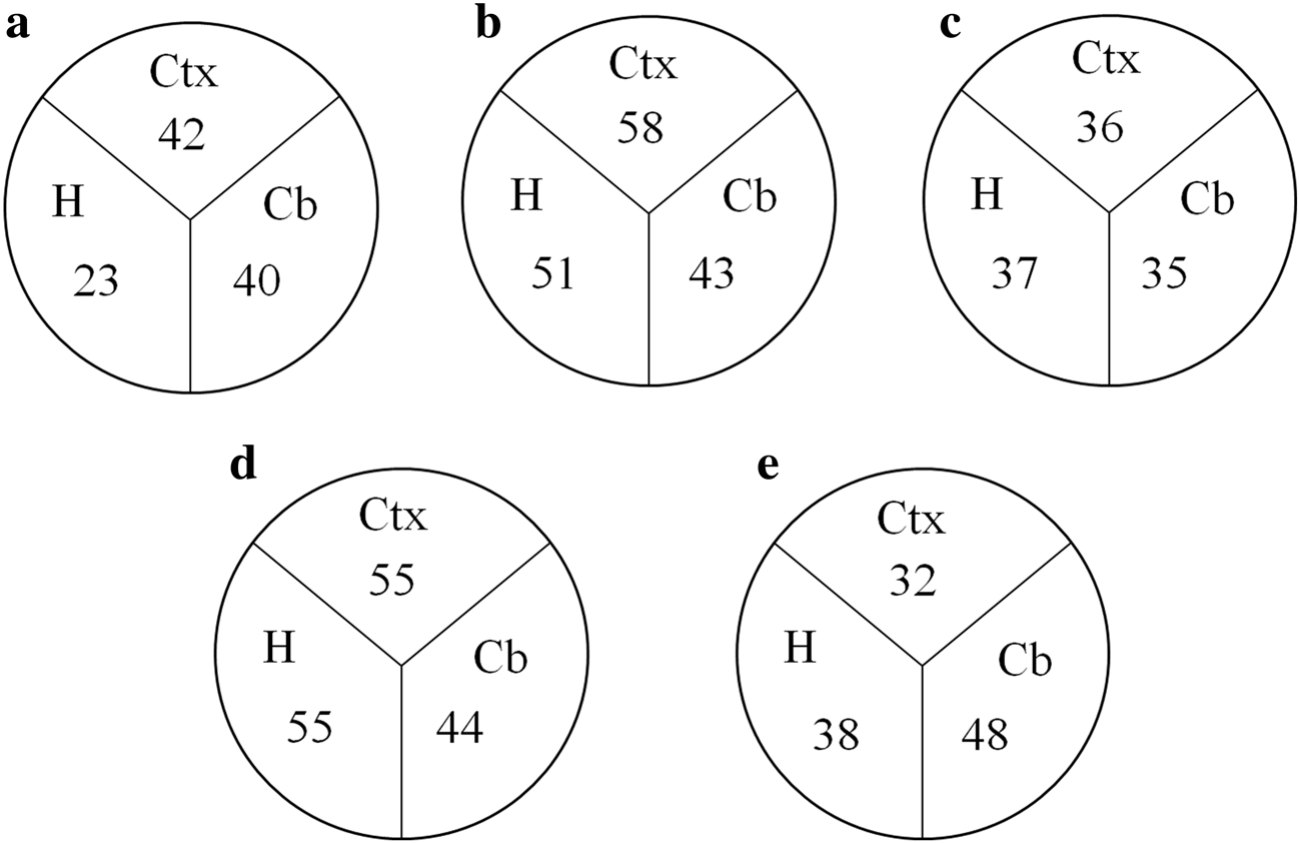
Diagrams showing changes in expression of proteins involved in redox state maintenance and regulation of cell death processes. Numerals in the sectors refer to the number of differentially expressed proteins in the cortex (Ctx), hippocampus (H) and cerebellum (Cb). The individual diagrams show the numbers of changes induced by maternal separation (S/C) in different brain regions of juvenile (**a**) and adult male (**b**) or female (**c**) rats or the comparison of differentially expressed proteins in brain tissue samples from males and females (M/F) of control (**d**) or maternally separated groups of rats (**e**). The complete list of differentially expressed proteins in three brain regions may be extracted from Additional file 5: Table S3 according to the color using filter tool. The number of differentially expressed proteins in a particular brain region is the sum of qualitative and quantitative changes in proteins with a null q-value and score higher than 4

Comparison of protein expression in different brain regions (Fig. 5) showed markedly fewer protein alterations induced by maternal separation (S/C) in hippocampus than in cortex and cerebellum from juvenile rats (Fig. 5a). The hippocampal proteome of juvenile rats was the least changeable, while the most dynamic changes were noticed in cortex from adult male rats (Fig. 5a and b). Moreover, the number of differentially expressed proteins in hippocampus was notably higher in adult male that in juvenile rats (Fig. 5a and b), suggesting that maternal separation has a markedly stronger impact on protein expression and metabolism in hippocampus, when compared to cortex or cerebellum, and may thus more profoundly affect functional properties of this brain area in adult male life. Another interesting dissimilarity arose in the cortex of adult male and female rats. While the number of differentially expressed proteins after maternal separation increased in adult male rats, compared to juvenile rats, it decreased in adult female rats (Fig. 5a–c). These data suggest that maternal separation induces sex-specific alterations in cerebrocortical proteome, male rats being affected to a wider extent.

There were several similarities in protein alterations induced by prolonged maternal separation. Interestingly, the vast majority of similar changes in protein expression were qualitative changes, meaning that some proteins were either detected or not detected in different compared samples. The similarities in differentially expressed proteins between the three brain regions for each age/sex group are summed up in Table 1. There were a higher number of similarities in differentially expressed proteins in samples from male (12) and female (14) than juvenile (8) rats. The expression of 4 proteins was changed in the same direction in all three brain regions for each age/sex experimental group, i.e. 85/88 kDa calcium-independent phospholipase A2 and P2X purinoceptor 7 in juvenile rats, [pyruvate dehydrogenase (acetyl-transferring)] kinase isozyme 2 in adult male rats and ribonucleotide reductase M2 B in adult female rats (Table 1). Cortex and cerebellum of juvenile rats showed a similar number of changed proteins. Whereas a higher number of similarities were found in cortex and cerebellum or in hippocampus and cerebellum of adult male rats, protein changes in adult female rats induced by maternal separation were more similar in cortex and hippocampus or in hippocampus and cerebellum. The CAP-GLY domain-containing linker protein 3 was altered in cortex and cerebellum in both adult male and female rats affected by maternal separation. Interestingly, this protein was detected only in maternally separated adult male rats and in control females (Additional file 5: Table S3). Table 2 presents the similarities in differentially expressed proteins between three age/sex groups in each of the three tested brain regions. The highest number of similarities (18) were found in cortex (Table 2), suggesting that the response induced by cell death stimuli in this brain area might be more similar than in hippocampus or cerebellum of juvenile, adult male and adult female rats. Two proteins were altered in the same directions concurrently in two brain regions and two age/sex group. The macrophage erythroblast attacher was detected only in cortex of control adult rats and in hippocampus of adult rats previously subjected to maternal separation. The scribbled planar cell polarity protein was present only in cortex of maternally separated juvenile and adult male rats, and it disappeared from hippocampus of maternally separated juvenile and adult male rats (Additional file 5: Table S3). The sum of similarities in differentially expressed proteins represents only a minor portion of the total number of changes, suggesting that maternal separation elicited age- and sex-dependent alterations in cell death-related proteins specific for individual brain regions.

**Table 1:** Proteins with similarly changed expression in the prefrontal cortex, hippocampus and cerebellum after prolonged maternal separation

Brain regions	Juvenile	Male	Female
Ctx/H/Cb	85/88 kDa calcium-independent phospholipase A2 [S] P2X purinoceptor 7 [S]	[Pyruvate dehydrogenase (acetyl-transferring)] kinase isozyme 2 [↓]	Ribonucleotide reductase M2 B [C]
Ctx/H		Apolipoprotein H [C] Tumor protein p53-binding protein, 2 [S]	Alpha-actinin-4 [S] Apoptosis regulator BAX [C] Beclin-1 [C] Cyclin dependent kinase inhibitor [C] Glutamate receptor ionotropic, delta-2 [C] PCI domain-containing 2 [C]
Ctx/Cb	ADP-ribosyl cyclase/cyclic ADP-ribose hydrolase 1 [S] Histidine-rich glycoprotein [C] Polyribonucleotide nucleotidyltransferase 1 [S] Protein kinase LYK5 [S]	Agrin [C] CAP-GLY domain-containing linker protein 3 [S] Glutamate receptor ionotropic, kainate 2 [S] NADH dehydrogenase [ubiquinone] 1 alpha subcomplex assembly factor 4 [S] RNA-binding protein 10 [S]	CAP-GLY domain-containing linker protein 3 [C] DNA topoisomerase 2 [S]
H/Cb	Calcipressin-2 [S] NADH dehydrogenase [ubiquinone] 1 alpha subcomplex assembly factor 4 [S]	Adenomatosis polyposis coli [C] Amino-terminal enhancer of split [C] Eph receptor B2 [S] FAS-associated death domain protein [C]	5-Demethoxyubiquinone hydroxylase [C] Aquaporin-1 [S] Craniofacial development protein 1 [C] Histidine-rich glycoprotein [S] Macrophage erythroblast attacher [S]

*Ctx* cortex, *H* hippocampuc, *Cb* cerebellum, [C], protein present exclusively in brain tissue of control rats; [S], protein present exclusively in brain tissue of maternally separated rats; [↓], protein downregulated after maternal separation

**Table 2:** Proteins with similarly changed expression in juvenile, adult male and female rats after prolonged maternal separation

Age/sex groups	Cortex	Hippocampus	Cerebellum
Juvenile/male/female	Glutamate receptor ionotropic, delta-2 [C]		Glutamate receptor ionotropic, NMDA 2C [S] Protein-tyrosine kinase 2-beta [C] TNF alpha-induced protein 8 [C]
Juvenile/male	ADP-ribosyl cyclase/cyclic ADP-ribose hydrolase 1 [S] CAP-GLY domain-containing linker protein 3 [S] HIG1 domain family member 1A [S] Scribbled planar cell polarity protein [S] Signal transducer and activator of transcription [S] Tumor protein p53-binding protein, 2 [S]	Amino-terminal enhancer of split [C] Aquaporin-1 [C] Metabotropic glutamate receptor 4 [S] Protein kinase LYK5 [C] Scribbled planar cell polarity protein [C]	Agrin [C] Gap junction protein [S] Glutamate receptor ionotropic, NMDA 2A [S] Mitogen-activated protein kinase kinase kinase kinase [C] NADH dehydrogenase [ubiquinone] 1 alpha subcomplex assembly factor 4 [S]
Juvenile/female	Calcipressin-1 [C] Calpastatin [S] RNA-binding protein 10 [C]	HIG1 domain family member 1A [C] NADH dehydrogenase [ubiquinone] 1 alpha subcomplex assembly factor 4 [S] RCG50226 [C]	ADP-ribosyl cyclase/cyclic ADP-ribose hydrolase 1 [S] Ceramide synthase 1 [S]
Male/female	Alpha-actinin-4 [S] Dedicator of cytokinesis 7 [C] DNA topoisomerase 2 [S] FAS-associated death domain protein [S] Macrophage erythroblast attacher [C] Metabotropic glutamate receptor 4 [C] Peptidyl-prolyl cis–trans isomerase FKBP1B [C] Protein Mpv17 [C]	Beclin-1 [C] Cyclin dependent kinase inhibitor [C] Macrophage erythroblast attacher [S] Retinal dehydrogenase 2 [S]	Cyclic AMP-responsive element-binding protein 1 [C]

[C], protein present exclusively in brain tissue of control rats; [S], protein present exclusively in brain tissue of maternally separated rats

### Sex- and age-related differences in proteomic profiles of maternally separated rats

A comparison of reversely expressed proteins by maternal separation at least in two brain regions revealed a larger number of changes in the adult male group (19 alterations) than in the adult female group (8 alterations) and the juvenile group (8 alterations). Inter-regional correlation between these changes in protein expression was noticed in cortex and hippocampus of the male adult group (10 alterations) or the juvenile group (5 alterations) (Additional file 5: Table S3).

When comparing the numbers of differentially expressed proteins in different brain regions of maternally separated adult male and female rats with corresponding controls (pairwise comparison of male and female rats), we noticed certain differences especially in cortex and hippocampus. The number of protein differences between samples from control male and female rats was markedly higher than the number of differences between maternally separated male and female rats (Fig. 5d and e), meaning that the sum of differences in protein expression between males and females were equalized by maternal separation whilst only a more limited number of other proteins were deregulated by this unfavorable intervention.

In animals subjected to maternal separation, 40 and 39 differences in protein levels between males and females ceased to exist in prefrontal cortex and hippocampus, respectively, while only 29 differences disappeared in cerebellum (Additional file 6: Table S4). On the other hand, there were 17, 22 and 33 sex-specific differences in protein levels incurred by maternal separation in cortex, hippocampus and cerebellum, respectively (Additional file 7: Table S5). Prolonged maternal separation of rat pups has similar impact on the expression of proteins implicated in the regulation of cell death and redox balance in both adult rat cortex and hippocampus, suggesting that these two brain structures display similar features with regard to their sensitivity to early postnatal stress in males and females, in contrast to the cerebellum. Nevertheless, a more detailed examination of sex differences in protein levels in different brain regions showed that protein expression changes to a great extent were unique to each brain region. In the group of differentially expressed proteins between sexes, only three proteins were simultaneously deregulated in the same direction in two brain regions after maternal separation. One protein, PCI domain-containing 2, disappeared from the cerebral cortex and hippocampus of previously maternally separated adult female rats. Another two proteins, FAS-associated death domain protein (FADD) and protein S100-B, were simultaneously deregulated in hippocampus and cerebellum. The adaptor protein FADD became undetectable in both hippocampus and cerebellum of adult male rats affected by maternal separation, while the level of this protein remained unaltered in females. Maternal separation resulted in a mild downregulation of S100-B in both hippocampus and cerebellum of male rats and led to a mild upregulation of this protein in the same regions of the female brain. These observations together suggest that maternal separation can eliminate naturally occurring sex differences in the expression profiles of proteins involved in controlling the cell death processes and maintaining oxidative stress balance in different brain regions and thus set slightly different conditions that may potentially modify the cellular responses to stimuli causing oxidative stress and cell death.

By comparing the alterations induced by maternal separation in protein expression between different brain regions in rat groups sorted according to age and sex, we found that 72 to 81 percent of protein changes in each rat group occurred only in one brain region. These findings suggest that most changes were age- and brain region-dependent and that maternal separation in the early stage of life may affect different processes in specific brain regions. We observed that maternal separation had a broad impact on the protein profiles of adult rat brains. Interestingly, these effects markedly differed from those observed in juvenile rats and were sex-dependent. It is known that ELS causes sex-specific responses, which often last into adulthood. Such sex differences might arise from sex-specific epigenetic regulation of gene expression during development, action of gonadal hormones (i.e., estradiol and testosterone) or microglia-mediated mechanisms [32, 47, 48]. ELS and maternal care significantly affect epigenetic modulation of various promoter regions, including promoter region of ERα gene [3]. Sex differences in the methylation status of the ERα promoter and ERα expression within the developing preoptic area may be partly caused by estradiol exposure associated with maternal care. Likewise, testosterone is believed to act via epigenetic processes, mainly histone acetylation, in the bed nucleus of the stria terminalis [49]. It can be assumed that similar epigenetic modifications of other promoter regions with subsequent altered expression of proteins involved in cell death might be employed in other brain areas. Maternal deprivation was shown to affect the level of estradiol in adult rats (PND 90) in a sex-specific manner and decrease the level of testosterone in adult male rats [50, 51], suggesting that maternal separation may cause dysregulation of gonadal hormones persisting into adulthood. The gonadal hormones have not only an impact on epigenetic modifications but also on expression of apoptotic and anti-apoptotic proteins, caspase activation, autophagy, mitochondrial membrane permeability, the number of microglia and their morphology, ROS production or handling of calcium related cellular signaling in neurons [47, 48, 52, 53]. Therefore, the levels of these hormones are key factors determining sex-specific cell death and other associated cellular processes at least in some brain regions. In response to harmful stimuli, female and male neurons have a tendency to undergo a caspase-dependent and caspase-independent cell death, respectively [54]. It is difficult to estimate if the change in testosterone level induced by maternal separation has a demasculinizing effect in relation to cell death process. In rodents, the critical period for masculinization of the brain begins at embryonic day 16 and ends within hours after birth [48], indicating that sex-specific patterns of cell death may be maintained or restructured into new sex-specific arrangements. In conclusion, despite the same experimental conditions during maternal separation for rats of both sexes, the adaptive response to maternal separation carried out during the first three postnatal weeks is sex-specific, possibly dependent on sex-specific levels of gonadal hormones and epigenetic modifications.

### Prolonged maternal separation and brain oxidative stress balance and apoptosis

The levels of glutathione, protein carbonyls, malondialdehyde and lipid hydroperoxides were determined in selected brain regions of both juvenile and young adult rats in order to explore the possible effect of maternal separation on redox balance. We did not find any significant differences between the levels of all these markers in brain tissue samples from control rats and those subjected to maternal separation (Additional file 8: Fig. S3). There was only a slight tendency towards higher MDA content in prefrontal cortex of maternally separated juvenile rats. Activity of glutathione peroxidase did not markedly differ between brain tissue samples from control and maternally separated rats (Additional file 9: Fig. S4).

In the next set of experiments, we determined expression of selected proteins (Bak, Bax, Bcl-XL, Bid, caspase 3, 8 and 12) involved in apoptotic processes. LFQ allows analyzing protein expression levels but not their activity. Therefore we used Western blotting to find out whether some proteins involved in apoptosis were activated by maternal separation. This approach was basically not used for validating the results of proteomic analysis. On the contrary, we mainly focused on selected apoptotic proteins which were not detected by mass spectrometry apparently because of their low abundance (Bak, Bcl-XL, Bid, caspase-8, and caspase-12) in order to extend the list of detected apoptotic proteins and deepen our knowledge about possible induction of apoptosis by maternal separation. Our Western blot analysis did not reveal any significant changes in the level of most of these proteins (Additional file 10: Fig. S5). Interestingly, only the levels of Bax, caspase-3 and Bcl-XL were decreased and Bid increased in cerebellum of juvenile rats subjected to maternal separation (Additional file 10: Fig. S5A). The members of the Bcl-2 family interact with each other to regulate apoptosis and mitochondrial outer membrane permeabilization (MOMP). Bid is activated by caspase-mediated cleavage into three fragments (p15, p13, and p11). The truncated p15 Bid (tBid) recruits inactive Bax and activates it allowing its insertion into mitochondrial membrane, which leads to Bak oligomerization and permeabilization of the mitochondrial outer membrane, resulting in a release of intermembrane space proteins and triggering apoptosis. Bcl-XL can bind to tBID and prevent Bax activation and oligomerization. Bcl-XL also binds to activated Bax [55, 56]. Besides the cerebellum of maternally separated juvenile rats, the levels of Bid in cerebellum of juvenile and adult rats were lower than those in cortex and hippocampus (Additional file 10: Fig. S5). We also detected cleaved protein fragments which may correspond to p15, p13, and p11 fragments. Their levels were higher in cerebellum than in cortex and hippocampus but they diminished in the cerebellum of maternally separated juvenile rats. This suggests that Bid is cleaved to a larger extent in cerebellum when compared with cortex and hippocampus and that this cleavage is suppressed by prolonged maternal separation in juvenile rats (Additional file 10: Fig. S5A). The relative abundance of Bcl-2 proteins is one of the key factors determining which binding interactions between Bcl-2 family members dominate and whether or not the cell will undergo MOMP committing it to apoptosis [56]. Whereas the levels of Bid binding partners, Bax and Bcl-XL, decreased in cerebellum of maternally separated juvenile rats, the level of Bak, which oligomerizes with Bax, was not altered (Additional file 10: Fig. S5A). Because Bax is pro-apoptotic and Bcl-XL anti-apoptotic, prolonged maternal separation apparently did not influence the extent of apoptosis. Notwithstanding, the observed decrease in cleavage of Bid induced by maternal separation may have a modulatory effect on the interactions with Bid binding partners and potential permeabilization of the mitochondrial outer membrane. Interestingly, a slight but statistically significant decrease in Bid level induced by maternal separation was found in hippocampus of adult female rats (Additional file 10: Fig. S5C). Curiously, there were no significant changes in the levels of Bid binding partners. We also noted increased expression of procaspase 3 in prefrontal cortex and hippocampus of maternally separated adult male rats (Additional file 10: Fig. S5B). However, there were no detectable cleaved forms of caspase 3 suggesting that this caspase was not activated. Likewise, there were no detectable cleaved forms of caspase 8 and 12 and maternal separation did not affect expression of these two procaspases in brain tissue of juvenile or adult rats (Additional file 10: Fig. S5). The comparison of these results with those from mass proteomic analysis is discussed below.

Some studies have indicated that maternal separation or deprivation may cause oxidative stress [26–29] and affect brain cell death [36, 57–63]. It is known that these two cellular processes are related to each other [16, 20, 64]. Oxidative stress plays a substantial role in neuronal cell death and neurodegeneration, which is the hallmark of various neurodegenerative diseases [24]. Among others, maternal separation is thought to induce epigenetic changes and may have implications for neurodevelopmental disorders [65]. Thus, one can assume that the molecular mechanisms implicated in the development of neuronal disorders as a result of maternal separation may include increased oxidative stress and cell death. Therefore, here we focused our attention on proteins potentially participating in these processes.

Our investigations revealed a higher correlation between differentially expressed proteins involved in maintaining redox balance and controlling cell death in the brain of adult female rats and indicated that maternal separation can induce similar changes in different brain areas. Distinct proteomic profiles identified in different brain regions of maternally separated rats can reflect different ability to cope with oxidative stress and resist cell death.

### The impact of maternal separation on proteins involved in the regulation of brain redox balance

There were only slight and sporadic changes in the expression levels of proteins involved in the regulation of redox balance in brain of maternally separated rats (Additional file 4: Table S2). The enzyme antioxidant system was apparently not substantially altered. Maternal separation did not affect levels of superoxide dismutases (SOD1, SOD2), catalase, two isozymes of glutathione peroxidase (GPx1, GPx4), peroxiredoxins (Prdx2, Prdx3, Prdx4, Prdx5, Prdx6) and thioredoxins (Txn1, Txn2, Txnl1 and Txndc17). Interestingly, only glutaredoxin 2 disappeared from prefrontal cortex of juvenile rats and from cerebellum of adult male rats, and it appeared in hippocampus of juvenile rats subjected to maternal separation (Additional file 4: Table S2). One of the functions of glutaredoxin 2 is to protect cells from oxidative stress-induced apoptosis. Glutaredoxin 2 overexpression protected human lens epithelial cells against H_2_O_2_-induced apoptosis while glutaredoxin 2 knockdown showed the opposite effect [66]. Expression profiles of enzymes known to catalyze reactions influencing the state and metabolism of antioxidant enzymes (thioredoxin reductases) and glutathione (glutathione reductase, glutathione transferases, glutamate-cysteine ligase, glutathione synthetase, hydroxyacyl glutathione hydrolase and lactoyl glutathione lyase) were not affected by maternal separation.

It has been demonstrated that the changes observed in expression of particular proteins in proteomic studies need not correlate with their activity [67, 68]. Although we detected only slight changes in proteins involved in the regulation of redox balance, it did not mean that maternal separation did not evoke alterations in redox homeostasis. Additionally, we also aimed to determine whether maternal separation could induce oxidative stress in different brain regions. Interestingly, we did not find any statistically significant changes in the levels of important markers of oxidative stress (glutathione, protein carbonyls, lipid peroxides and hydroperoxides) in different brain regions of both juvenile and young adult rats. Activity of glutathione peroxidase in different brain regions of maternally separated rats was also not changed.

The induction and intensity of oxidative stress in brain of maternally separated pups evidently depends on experimental conditions (the regimen and duration of separation), as well as on brain region, age and sex [26–30]. Our experiments showed that maternal separation for 3 h daily throughout the first three weeks of life did not induce oxidative stress either in prefrontal cortex or hippocampus or cerebellum of both juvenile and adult rats. Diehl et al. [30] who used similar experimental conditions (3-h daily separation for the first ten postnatal days), did not find any changes in the activity of antioxidant enzymes in the hippocampus of adult rats. These data are consistent with the results of our present study and suggest that the prolonged maternal separation does not induce oxidative stress in rat brain. It is imaginable that short-term maternal separation may be associated with the induction of oxidative stress, whereas prolonged maternal separation can lead to restoration of the initial state of the antioxidant defense system.

### The impact of maternal separation on proteins involved in cell death processes

Maternal separation had a large impact on the proteome associated with cell death (Additional file 4: Table S2). The observed alterations in protein levels appeared to be brain region-, age- and sex-dependent. For preparing a clear overview of differentially expressed proteins we selected those with fundamental roles in different types of cell death or those associated with the processes occurring during cell death. Protein alterations induced by maternal separation and comparison of protein levels between adult male and female rats in control or separated groups were sorted out according to their occurrence in cortex (Table 3), hippocampus (Table 4) and cerebellum (Table 5).

**Table 3:** A list of differentially expressed proteins in the prefrontal cortex with fundamental roles in the regulation of cell death

Protein ID	Gene name	Protein name	J S/C	M S/C	F S/C	C M/F	S M/F	Protein function
F1LMM8 Q64536	Pdk2	[Pyruvate dehydrogenase (acetyl-transferring)] kinase isozyme 2, mitochondrial	–	↓ (0.35)	–	–	–	p53 activation decreases Pdk2 transcription [92]
P97570	Pla2g6	85/88 kDa calcium independent phospholipase A2	S	–	–	–	–	tBID and Bax augments Pla2g6 (iPLA2) activity via ROS production leading to changes in the MOM [93] Overexpression of iPLA2 increased the rate of apoptosis, iPLA2 is cleaved by caspase-3 [94]
								β-cell apoptosis is attributable to the modulation of 5′SS selection in Bcl-X pre-mRNA by bioactive lipids modulated by iPLA2 [95]
G3V8T9 Q9JKL3 Q63690	Bax	Apoptosis regulator BAX	–	N	C	F	–	Pro-apoptotic member of Bcl-2 family, its activation permeabilizes the MOM during apoptosis [96] Deletion of Bax is sufficient to inhibit apoptosis [97] Bax is cleaved by active calpains [80]
A0A0G2JZS5 Q7TS62 P53563 E9PU78	Bcl2l1	Bcl-2-like protein 1	C	–	–	–	–	Bcl2l1 (Bcl-X) gene generates two protein products by alternative pre-mRNA splicing: Bcl-X_L_ is anti-apoptotic, while Bcl-X_S_ is pro-apoptotic; deletion of Bcl-X in mice results in neuronal death in the brain during late embryonic development; Bcl-X_L_ overexpression attenuates brain injury in neonatal rodents [77]
Q91XJ1	Becn1	Beclin-1	–	N	C	F	N	Beclin-1-induced autophagy is inversely correlated with apoptosis [13]
Q6IN33	Rcan1	Calcipressin-1	C	N	C	F	N	Calcineurin regulation; Rcan1 absence enhances calcineurin activity and Fas ligand expression [98]
F1LPH1 P27321 D3ZL24	Cast	Calpastatin	S	N	S	N	F	Cast is a specific endogenous inhibitor of calpains and is cleaved by caspases [99]
P55213	Casp3	Caspase-3	–	S	C	F	M	Ceavage and activation of caspase-3 initiates apoptosis; essential for neuronal cell death [100] Caspase-3 expression is regulated with age [21] A regulatory calpain/caspase-3 cross-talk [101]
Q1HL14	Cers1	Ceramide synthase 1	–	S	N	N	M	Inhibition of de novo ceramide synthesis inhibited caspase 3/7 activation and apoptosis [102] Overexpression of CerS1 and increased level of C18 ceramide resulted in activation of ER stress and inhibition of cell viability, independent of Bax [103]
Q5RK17 D4A7Z5	Diablo LOC100360940	Diablo homolog	S	C	–	–	F	Diablo is located in the intermembrane space of mitochondria and is released into the cytosol during apoptosis and thereby enabling the activation of caspases [104]
Q8R2E7	Fadd	FAS-associated death domain protein	–	S	S	N	–	Pro-apoptotic adapter protein involved in extrinsic pathway of apoptosis [100] Overexpression of FADD promotes apoptosis [104]
Q63226 F1LXB6	Grid2	Glutamate ionotropic receptor, delta-2	C	C	C	↓ (0.47)	–	Gain-to-function mutation of Grid2 induces neuronal death [105]; this mutation is associated with increased expression of Bax, Bcl-X_S_, caspase-3 and -8 [106] TNF-α has a role in regulation of Grid2 gene expression, which can be a suppressor in TNF-induced neurodegeneration [107]
P42260 F1M855	Grik2	Glutamate ionotropic receptor, kainate 2	N	S	–	F	–	Grik2 (GluR6) promotes Bax translocation and increase in caspase-3 activation [108]
Q8VH49	Higd1a	HIG1 domain family 1A, mitochondrial	S	S	N	N	M	Higd1a is a survival factor and is associated with caspase-3 activation [109, 110]
F1MAL5	Irs2	Insulin receptor substrate 2	N	C	N	M	N	CaMK4/CREB/IRS2 cascade can inhibit apoptosis [111] IRS2 knockout increased cell death and activation of caspase-3 and -8 as well as the levels of Fadd, Bcl-2, Bcl-X_L_ and p53 [112]
P31423	Grm4	Metabotropic glutamate receptor 4	–	C	C	–	N	Decreased expression of GRM4 gene is associated with apoptosis of cerebellar granule neurons [86] Activation of Grm4 can activate pro-caspase 8/9/3 and disrupt the balance of Bcl-2/Bax expression [113]
P06907	Mpz	Myelin protein P0	N	C	N	M	N	Mpz knockdown induced apoptosis [114]
A0A0G2JZT0 Q64663	P2rx7	P2X purinoceptor 7	S	↓ (0.39)	–	↑ (3.28)	–	P2rx7s mediate caspase-8 and caspase-3 dependent apoptosis [115] Its activation is associated with Ca^2+^ responses and TNF-α production [116–118]
F1LNG8 Q5BK62	Mpv17	Protein Mpv17	S	C	C	–	N	The increase of Mpv17 expression can be accompanied by the enhanced expression of p53, Bax, cyt c and active caspase-3 and decreased expression of Bcl-2 in the pathological proces [119]
B1WC67	Slc25a24	RCG29001	–	–	S	–	F	A survival factor, knock-down of Slc25a24 led to reduction of Ca^2+^ buffering capacity and sensitized cellts to cell death induced by mitochondrial permeability transition [120]
Q63259	Ptprn	Receptor–type tyrosine protein phosphatase-like N	–	–	C	–	M	Ptprn knock-down can prevent apoptosis [121]
Q63639	Aldh1a2	Retinal dehydrogenase 2	N	–	–	↓ (0.36)	–	Aldh1a2 mediates conversion of retinol into active retinoic acid altering the expression of Bcl-2, Bax, Bid, caspase-8 and -3 [122]
D4ADQ1	Rrm2b	Ribonucleotide reductase M2 B (TP53 inducible)	N	S	C	F	M	Loss of Rrm2b can increase apoptosis [123] Rrm2b expression is induced by stress [124] Rrm2b cleavage is mediated by caspase-8 and -3 [125]
P70501 A0A0G2K3S6	Rbm10	RNA-binding protein 10	C	S	C	F	M	Rbm10 knock-down can decrease caspase activation [126] Rbm10 regulates alternative splicing of Fas and Bcl-X genes [127] Rbm10 expression is associated with increased apoptosis [128]
A0A0G2JXI4 P52632	Stat5b;Stat5a	Signal transducer and activator of transcription	S	S	N	N	M	Stat5 regulate expression of Bcl-2 and Bcl-X_L_ and the level of caspase-3 [129–131]
D4A9G3 A0A0G2K2P3	Tnfaip8	TNF-α induced protein 8	N	S	–	F	–	Tnfaip8 can suppress TNF-mediated apoptosis by inhibiting TNF-induced caspase-8 activity [69]
F1M5H6 D4A6A8 A0A0G2KB61	Tp53bp2	Tumor protein p53-binding protein 2	S	S	–	F	–	Tp53bp2 binds to p53 and Bcl-2; it can alter p53 protein conformation and enhance the binding activity of p53 to the promotes of Bax [132]

*C* protein detected only in samples of the cerebral cortex from control rats, *S* protein detected only in samples of the cerebral cortex from maternally separated rats, *M* protein detected only in samples of the cerebral cortex from adult male rats, *F* protein detected only in samples of the cerebral cortex from adult female rats, *N* protein not detected in the samples, –, unchanged protein expression between the samples; ↓, protein down-regulated in samples of the cerebral cortex from maternally separated rats; ↑, protein up-regulated in samples of the cerebral cortex from maternally separated rats

**Table 4:** A list of differentially expressed proteins in the hippocampus with fundamental roles in the regulation of cell death

Protein ID	Gene name	Protein name	Y S/C	M S/C	F S/C	C M/F	S M/F	Protein function
F1LMM8 Q64536	Pdk2	[Pyruvate dehydrogenase (acetyl-transferring)] kinase isozyme 2, mitochondrial	–	↓ (0.46)	–	–	–	p53 activation decreases Pdk2 transcription [92]
P97570	Pla2g6	85/88 kDa calcium independent phospholipase A2	S	–	C	–	M	tBID and Bax augments Pla2g6 (iPLA2) activity via ROS production leading to changes in the MOM [93] Overexpression of iPLA2 increased the rate of apoptosis, iPLA2 is cleaved by caspase-3 [94] β-cell apoptosis is attributable to the modulation of 5′SS selection in Bcl-X pre-mRNA by bioactive lipids modulated by iPLA2 [95]
G3V8Q9 A0A0G2K8G0 P70478	Apc	Adenomatosis polyposis coli	–	C	–	–	F	APC can regulate apoptosis by governing the level of caspase-3 [133]
P23928	Cryab	Alpha-crystallin B chain	C	–	–	–	–	Cryab prevents apoptosis; it binds to caspase-3 [134], Bax and Bcl-X_S_ [135] and p53 [136] Cryab gene is a targer of p53 and p53-dependent apoptosis is affected by Cryab expression [137]
G3V8T9 Q9JKL3 Q63690	Bax	Apoptosis regulator BAX	–	S	C	F	M	Pro-apoptotic member of Bcl-2 family, its activation permeabilizes the MOM during apoptosis [96] Deletion of Bax is sufficient to inhibit apoptosis [97] Bax is cleaved by active calpains [80]
E9PST5	Acin1	Apoptotic chromatin condensation inducer 1	–	↑ (2.02)	↓ (0.36)	–	↑ (3.30)	Acin1 can induce apoptotic chromatin condensation after its cleavage by caspase-3 [137]
Q91XJ1	Becn1	Beclin-1	N	C	C	–	N	Beclin-1-induced autophagy is inversely correlated with apoptosis [13]
Q6IN33	Rcan1	Calcipressin-1	–	C	S	M	F	Calcineurin regulation [138]; Rcan1 absence enhances calcineurin activity and Fas ligand expression [98]
A0A0G2JSK3 Q8CH27	Rcan2	Calcipressin-2	S	N	S	N	F	Calcineurin regulation [138]
P48199 A0A0G2K8V5	Crp	C-reactive protein	C	–	–	–	–	Crp can induce the upregulation of p53 and increase caspase-3 activity [139]
F1MAJ0 A0A0G2JY48	Ephb2	Eph receptor B2	–	S	C	F	M	Ephb2 can prevent against Fas-triggered apotosis by inhibition of caspase-8 and caspase-3 [140]
Q8R2E7	Fadd	FAS-associated death domain protein	S	C	–	–	F	Pro-apoptotic adapter protein involved in extrinsic pathway of apoptosis [100] Overexpression of FADD promotes apoptosis [103]
Q63226 F1LXB6	Grid2	Glutamate ionotropic receptor, delta-2	N	N	C	F	–	Gain-to-function mutation of Grid2 induces neuronal death [105]; this mutation is associated with increased expression of Bax, Bcl-X_S_, caspase-3 and -8 [106] TNF-α has a role in regulation of Grid2 gene expression, which can be a suppressor in TNF-induced neurodegeneration [107]
G3V913 P42930	Hspb1	Heat shock 27 kDa	–	↑ (2.14)	↑ (2.39)	–	–	Hspb1 indirectly suppresses stress-induced Bax oligomerization and translocation to mitochondria; it can inhibit caspase-3 activity [141] Hspb1 can mediate Bad inactivation [142]
Q8VH49	Higd1a	HIG1 domain family 1A, mitochondrial	C	N	C	F	N	Higd1a is a survival factor and is associated with caspase-3 activation [109, 110]
P31423	Grm4	Metabotropic glutamate receptor 4	S	S	N	N	M	Decreased expression of GRM4 gene is associated with apoptosis of cerebellar granule neurons [86] Activation of Grm4 can activate pro-caspase 8/9/3 and disrupt the balance of Bcl-2/Bax expression [113]
A0A0G2JZT0 Q64663	P2rx7	P2X purinoceptor 7	S	–	–	–	–	P2rx7s mediate caspase-8 and caspase-3 dependent apoptosis [115] Its activation is associated with Ca^2+^ responses and TNF-α production [116–118]
P27008	Parp1	Poly [ADP-ribose] polymerase 1	–	C	–	–	F	Parp1 is cleaved by caspase-3 [21]
F1LNG8 Q5BK62	Mpv17	Protein Mpv17	–	N	–	F	F	The increase of Mpv17 expression can be accompanied by the enhanced expression of p53, Bax, cyt c and active caspase-3 and decreased expression of Bcl-2 in the pathological proces [119]
P70600	Ptk2b	Protein-tyrosine kinase 2-beta	–	↓ (0.49)	–	–	–	Overexpression of Ptk2b can induce apoptosis [143]
B1WC67	Slc25a24	RCG29001	S	–	C	–	M	A survival factor, knock-down of Slc25a24 led to reduction of Ca^2+^ buffering capacity and sensitized cellts to cell death induced by mitochondrial permeability transition [120]
Q63639	Aldh1a2	Retinal dehydrogenase 2	N	S	S	N	–	Aldh1a2 mediates conversion of retinol into active retinoic acid, which the expression of Bcl-2, Bax, Bid, caspase-8 and -3 [122]
D4ADQ1	Rrm2b	Ribonucleotide reductase M2 B (TP53 inducible)	–	N	C	F	N	Loss of Rrm2b can increase apoptosis [123] Rrm2b expression is induced by stress [124] Rrm2b cleavage is mediated by caspase-8 and -3 [125]
P70501 A0A0G2K3S6	Rbm10	RNA-binding protein 10	–	N	–	F	F	Rbm10 knock-down can decrease caspase activation [126] Rbm10 regulates alternative splicing of Fas and Bcl-X genes [127] Rbm10 expression is associated with increased apoptosis [128]
A0A0G2JXI4 P52632	Stat5b;Stat5a	Signal transducer and activator of transcription	–	–	S	M	–	Stat5 regulate expression of Bcl-2 and Bcl-X_L_ and the level of caspase-3 [129–131]
F1M5H6 D4A6A8 A0A0G2KB61	Tp53bp2	Tumor protein p53-binding protein 2	–	S	N	N	M	Tp53bp2 binds to p53 and Bcl-2; it can alter p53 protein conformation and enhance the binding activity of p53 to the promotes of Bax [132]

*C* protein detected only in samples of the cerebral cortex from control rats, *S* protein detected only in samples of the cerebral cortex from maternally separated rats, *M* protein detected only in samples of the cerebral cortex from adult male rats, *F* protein detected only in samples of the cerebral cortex from adult female rats, *N* protein not detected in the samples, –, unchanged protein expression between the samples; ↓, protein down-regulated in samples of the cerebral cortex from maternally separated rats; ↑, protein up-regulated in samples of the cerebral cortex from maternally separated

**Table 5:** A list of differentially expressed proteins in the cerebellum with fundamental roles in the regulation of cell death

Protein ID	Gene name	Protein name	Y S/C	M S/C	F S/C	C M/F	S M/F	Protein function
F1LMM8 Q64536	Pdk2	[Pyruvate dehydrogenase (acetyl-transferring)] kinase isozyme 2, mitochondrial	–	↓ (0.41)	–	–	–	p53 activation decreases Pdk2 transcription [92]
P97570	Pla2g6	85/88 kDa calcium independent phospholipase A2	S	–	N	M	M	tBID and Bax augments Pla2g6 (iPLA2) activity via ROS production leading to changes in the MOM [93] Overexpression of iPLA2 increased the rate of apoptosis, iPLA2 is cleaved by caspase-3 [94] β-cell apoptosis is attributable to the modulation of 5′SS selection in Bcl-X pre-mRNA by bioactive lipids modulated by iPLA2 [95]
Q9JKL8	Adnp	Activity-dependent neuroprotector homeobox protein	–	–	C	–	M	Down-regulation of Adnp up-regulates p53 [144] NAP sequence of Adnp reduces activity of caspase-3 [145] Dysregulation of Adnp expression increases Bcl-2 expression in hippocampus [146]
G3V8Q9 A0A0G2K8G0 P70478	Apc	Adenomatosis polyposis coli	–	C	N	M	N	APC can regulate apoptosis by governing the level of caspase-3 [134]
P23928	Cryab	Alpha-crystallin B chain	S	↓ (0.50)	–	–	–	Cryab prevents apoptosis; it binds to caspase-3 [134], Bax and Bcl-X_S_ [135] and p53 [136] Cryab gene is a targer of p53 and p53-dependent apoptosis is affected by Cryab expression [136]
A0A0G2JZS5 Q7TS62 P53563 E9PU78	Bcl2l1	Bcl-2-like protein 1	–	–	N	M	M	Bcl2l1 (Bcl-X) gene generates two protein products by alternative pre-mRNA splicing: Bcl-X_L_ is anti-apoptotic, while Bcl-X_S_ is pro-apoptotic; deletion of Bcl-X in mice results in neuronal death in the brain during late embryonic development; Bcl-X_L_ overexpression attenuates brain injury in neonatal rodents [77]
Q91XJ1	Becn1	Beclin-1	N	–	N	M	M	Beclin-1-induced autophagy is inversely correlated with apoptosis [13]
A0A0G2JSK3 Q8CH27	Rcan2	Calcipressin-2	S	–	N	M	M	Calcineurin regulation [138]
F1LS29 P97571	Capn1	Calpain-1 catalytic subunit	N	S	N	N	M	Calpain is a Ca^2+^-dependent protease, which cleaves Bid, Bcl-2, Bcl-X_L_ [17] and Bax [80] It cleaves caspase-3, -8 and -12, p53 and NMDA receptors [81]
A0A0G2JYD8 G3V7U6 Q8R4C0	Capn5	Calpain-5	–	S	N	N	M	Ca^2+^-dependent protease
P55213	Casp3	Caspase-3	–	N	S	N	F	Cleavage and activation of caspase-3 initiates apoptosis; essential for neuronal cell death [100] Caspase-3 expression is regulated with age [21] a regulatory calpain/caspase-3 cross-talk [101]
Q1HL14	Cers1	Ceramide synthase 1	S	N	S	N	F	Inhibition of de novo ceramide synthesis inhibited caspase 3/7 activation and apoptosis [102] Overexpression of CerS1 and increased level of C18 ceramide resulted in activation of ER stress and inhibition of cell viability, independent of Bax [103]
P15337 A0A0G2K748	Creb1	Cyclic AMP-responsive element-binding protein 1	–	C	C	–	N	Enhanced Creb activity leads to increased Bcl-2 promoter activity and cell survival [147]
F1MAJ0 A0A0G2JY48	Ephb2	Eph receptor B2	-	S	–	F	–	Ephb2 can prevent against Fas-triggered apotosis by inhibition of caspase-8 and caspase-3 [140]
Q8R2E7	Fadd	FAS-associated death domain protein	–	C	–	–	F	Pro-apoptotic adapter protein involved in extrinsic pathway of apoptosis [100] Overexpression of FADD promotes apoptosis [103]
Q63226 F1LXB6	Grid2	Glutamate ionotropic receptor, delta-2	–	↑ (3.12)	–	–	–	Gain-to-function mutation of Grid2 induces neuronal death [105]; this mutation is associated with increased expression of Bax, Bcl-X_S_, caspase-3 and -8 [106] TNF-α has a role in regulation of Grid2 gene expression, which can be a suppressor in TNF-induced neurodegeneration [107]
P42260 F1M855	Grik2	Glutamate ionotropic receptor, kainate 2	–	S	–	F	–	Grik2 (GluR6) promotes Bax translocation and increase in caspase-3 activation [108]
Q62648 P35439	Grin1	Glutamate ionotropic receptor, NMDA 1	–	C	–	–	F	Up-regulation of Grin1 (NR1 subunit) is associated with increased expression of Bax and decreased expression of Bcl-X_L_ [148]
Q00959 G3V9C5	Grin2a	Glutamate ionotropic receptor, NMDA 2A	S	S	N	N	M	NMDARs containing Grin2a (NR2A subunit) promotes neuronal survival [149]; down-regulation of Creb was exaggerated in neurons over-expressing Grin2A [83]
G3V746 Q00960	Grin2b	Glutamate ionotropic receptor, NMDA 2B	C	S	N	N	M	NMDARs containing Gin2b (NR2B subunit) promotes neuronal death [149]; down-regulation of Creb was exaggerated in neurons over-expressing Grin2b [83]
A0A0G2JSH8 Q00961	Grin2c	Glutamate ionotropic receptor, NMDA 2C	S	S	S	N	–	NR2C expression supports neuronal survival [150]
O35821	Mybbp1a	Myb-binding protein 1A	–	–	C	–	M	Mybbp1a can increase Bax expression via p53 acetylation [151] Mybbp1a down-regulation induces apoptosis via caspase-3 activation [152]
P06907	Mpz	Myelin protein P0	C	↓ (0.34)	↑ (4.08)	↑ (7.98)	–	Mpz knockdown induced apoptosis [114]
A0A0G2JZT0 Q64663	P2rx7	P2X purinoceptor 7	S	–	–	–	–	P2rx7s mediate caspase-8 and caspase-3 dependent apoptosis [115] Its activation is associated with Ca2 + responses and TNF-α production [116–118]
F1LNG8 Q5BK62	Mpv17	Protein Mpv17	–	N	↑ (3.97)	F	F	The increase of Mpv17 expression can be accompanied by the enhanced expression of p53, Bax, cyt c and active caspase-3 and decreased expression of Bcl-2 in the pathological proces [119]
P70600	Ptk2b	Protein-tyrosine kinase2-beta	C	C	C	↑ (5.55)	N	Overexpression of Ptk2b can induce apoptosis [143]
D4ADQ1	Rrm2b	Ribonucleotide reductase M2 B (TP53 inducible)	N	N	C	F	N	Loss of Rrm2b can increase apoptosis [123] Rrm2b expression is induced by stress [124] Rrm2b cleavage is mediated by caspase-8 and -3) [125]
P70501 A0A0G2K3S6	Rbm10	RNA-binding protein 10	–	S	–	F	–	Rbm10 knock-down can decrease caspase activation [126] Rbm10 regulates alternative splicing of Fas and Bcl-X genes [127] Rbm10 expression is associated with increased apoptosis [128]
D4A280	Pak7	Serine/threonine-protein kinase PAK 5	–	S	C	F	M	Pak7 expression can prevent apoptosis by phosphorylating of Bad on Ser112 and inhibition of caspase-3 and PARP cleavage [153]
D4A9G3 A0A0G2K2P3	Tnfaip8	TNF-α induced protein 8	C	C	C	–	N	Tnfaip8 can suppress TNF-mediated apoptosis by inhibiting TNF-induced caspase-8 activity [69]

*C* protein detected only in samples of the cerebellum from control rats, *S* protein detected only in samples of the cerebellum from maternally separated rats, *M* protein detected only in samples of the cerebellum from adult male rats, *F* protein detected only in samples of the cerebellum from adult female rats, *N* protein not detected in the samples, –, unchanged protein expression between the samples; ↓, protein down-regulated in samples of the cerebellum from maternally separated rats; ↑, protein up-regulated in samples of the cerebellum from maternally separated rats

Here, we focused on two major types of programmed cell death: apoptosis and autophagy. Three signaling pathways participating in apoptosis have been described. The intrinsic pathway comprises caspase-9 and caspase-3, Bcl-2 family proteins and structural as well as functional changes in mitochondria. The extrinsic pathway is triggered by activation of death receptors (e.g., Fas receptor) or dependence receptors (e.g., netrin receptors). It is executed by caspase-8 via the formation of a molecular complex consisting of Fas receptor, FADD and caspase-8 [16]. TNFAIP8 protein (tumor necrosis factor, alpha-induced protein 8) suppressed TNF-α-mediated apoptosis by inhibiting TNF-α-induced caspase-8 enzymatic activity but not its processing [69]. The expression of some Bcl-2 family proteins and Fas receptor is regulated by the p53 tumor suppressor gene. The p53 protein alone is able to bind to Bcl-2 family proteins and modulates their function [70]. In recent years, yet another pathway has been proposed, whereby endoplasmic reticulum (ER) stress leads to direct activation of caspase-12 located at the ER [71]. The other type of cell death, autophagy, is mediated via insulin receptor, beclin-1 and Atg proteins. The calpain system negatively regulates autophagy and mediates the crosstalk between apoptosis and autophagy [13]. It seems that autophagy accompanies rather than promotes cell death and represents a failed survival attempt during the stress response [72]. Apoptosis may also be induced by excitotoxicity caused by excessive stimulation of the NMDA subtype of glutamate receptor [71].

### Apoptosis

We detected certain changes in levels of caspase-3, Bcl-2-like 1 protein and Bax, which are associated with the intrinsic apoptotic pathway. However, there were some discrepancies between the data from mass spectrometric and Western blot analyses. The protein levels detected by proteomic analysis did not always correlate with those determined by Western blotting. There are two possible explanations for these discrepancies. Firstly, one source of inconsistencies may have its origin in the detection limits of mass spectrometric quantification. For example, caspase-3 was detected at intensities from 22.69 to 24.19 in all three brain regions of juvenile rats by mass spectrometry but only in three from twelve adult brain samples at intensities from 21.89 to 22.72. It means that caspase-3 was expressed at lower level in adult rat brain and thus hardly detectable in our experimental conditions. In contrast to low abundance of caspase-3, the most abundant proteins (such as mitochondrial 60 kDa heat shock protein, elongation factor 1-alpha 2, fructose-bisphosphate aldolase, hemoglobin subunit beta-1, and serum albumin) were detected at intensities above 30 (Additional file 4: Table S2). Western blotting turned out to be more sensitive for detecting procaspase-3 because this protein was detected in all samples from adult brain by this method. Apart from cerebellum of maternally separated juvenile rats, the levels of procaspase-3 detected in all three regions of adult brain were overall lower than in samples from juvenile rats. Similar observations were made for Bax protein. In this way, results from Western blot analyses may support the correctness of proteomic data. Secondly, it is known that proteins involved in apoptosis, such as Bcl-2 family members and caspases, are regulated by posttranslational modifications, e.g. phosphorylation of Bax and caspases, polyubiquitination of Bax and caspases, and N-acetylation and S-nitrosylation of caspases [56, 73]. Bcl-2 proteins and caspases are cleaved into active fragments and Bcl-2 proteins form homooligomers or interact with other members of the family to form heterodimers [56, 74, 75]. The different molecular forms of one protein (posttranslational modifications, active fragments and oligomers) have changed electrophoretic mobility and are detected as separate bands by electrophoresis and Western blotting. Besides that, the number of protein molecular forms, antibody reactivity and accessibility of epitopes to which antibodies bind, are key factors determining which molecular forms of proteins will be detected by Western blotting. This is why more than a single band may appear on a blot when analyzing Bcl-2 proteins and caspases. For example, caspase-3 was detected as a 30-kDa band and also a 60-kDa band (presumably a dimer) on some Western blots. Anti-caspase-3 antibody apparently exhibited preferential reactivity against the 30-kDa form of the enzyme and Western blotting based on using this antibody provided more sensitive detection of this protein than LFQ. Bak and Bid were detected as monomers as well as bands with higher molecular weight, which could correspond to dimers or posttranslational modifications of these proteins. It can be assumed that Western blotting depending on antibody reactivity allows a sensitive detection of some molecular forms of a certain protein. On the contrary, protein levels determined by mass spectrometry can obviously include several molecular forms of one protein and low abundant proteins may thus be less detectable by LFQ when compared with Western blotting. In this way, the protein levels determined by Western blotting are not always directly comparable with those determined by mass spectrometry.

Bcl-2-like 1 protein disappeared from cortex of juvenile rats affected by maternal separation. This protein and Bcl-XL determined by Western blotting are two from several isoforms produced by the *BCL2L1* gene [76, 77]. Bcl-2-like 1 protein was detected by mass spectrometry as a low-abundant protein with four IDs in the Uniprot database (A0A0G2JZS5; Q7TS62; P53563; E9PU78) with different molecular weights and numbers of amino acids. Bcl-XL appeared as two bands at an approximate molecular weight of 26 kDa on Western blots, which could represent its unmodified and phosphorylated forms [78] and/or the long isoform Q7TS62 with higher molecular weight. The disappearance of Bcl2-like 1 protein in cortex of maternally separated juvenile rats compared to control rats could be associated with changed levels of other isoforms than Bcl2-XL produced by the *BCL2L1* gene. The decrease in the level of Bcl-XL in cerebellum of maternally separated juvenile rats had little impact on overall expression of different isoforms produced by *BCL2L1*.

Caspase-3 and Bax were found to be differentially expressed in brain tissue samples from adult rats. Interestingly, greater changes were observed in samples from female than male rats. The expression level of caspase-3 in control adult rats was generally lower than in juvenile animals (Tables 3, 4 and 5). This finding corresponds well to the notion that the executioner caspases (e.g., caspase-3) are downregulated with aging [15], which may be associated with the reduced propensity of neurons to initiate apoptosis in later development [18]. Our data are consistent with this notion; caspase-3 was detected in all three control brain tissue samples of juvenile rats but only in the cortex of adult female and not male rats (Additional file 5: Table S3). Maternal separation upregulated the level of caspase-3 in the male cortex, as determined by both LFQ and Western blot analysis (Table 3 and Additional file 9: Fig. S5). Caspase-3 was also present in cortex of control adult females and it appeared in cerebellum of maternally separated adult female rats (Tables 3 and 5). Alterations of caspase-3 in juvenile and female rat brain did not correlate with the results of Western blotting (Additional file 5: Table S3 and Additional file 10: Fig. S5). It is worth mentioning here that caspase-3 detected by mass spectrometry might include the non-active form, active fragments and dimers of the enzyme while caspase-3 determined by Western blotting represent only the non-active form of the enzyme. The expression of Bax in cortex and hippocampus of control male rats was lower than its expression in the respective brain regions of control juvenile and adult female rats. Interestingly, maternal separation did not affect the expression of Bax in juvenile rats (Tables 3 and 4). This protein appeared in hippocampus of adult male rats and disappeared from this brain structure of adult female rats subjected to previous maternal separation (Table 4). The changes in Bax expression determined by mass spectrometry did not correlate with those detected by Western blotting, mainly in adult rat brain. However, it should be noted here that it does not seems appropriate to compare the results obtained by these two methods because Bax detected by mass spectrometry includes three protein isoforms, two long isoforms with the length of 192 amino acids and molecular weight 21 kDa (IDs in the Uniprot Database are Q63690 and G3V8T9) and one short isoform with the length of 173 amino acids and molecular weight 19 kDa (ID Q9JKL3). Long and short isoforms are expected to be resolved by SDS-PAGE, but we were able to detected only one 20-kDa band. Two long isoforms of Bax differ in three amino acids at positions 6, 7, and 36 and this may possibly affect its recognition and detection by specific antibody. The distinct alterations in the expression of caspase-3 and Bax induced by maternal separation suggest that this type of early postnatal stress may elicit sex-specific differences in brain susceptibility to cell death. The impact of maternal separation on activation of the intrinsic apoptotic pathway was assessed by determining the cleavage of caspase-3. Western blot analysis of brain tissue samples revealed the presence of mainly uncleaved procaspase-3 (molecular weight 30 kDa) (Additional file 10: Fig. S5). Another band around 60 kDa most likely belonged to a dimer of the enzyme. These data indicate that the apoptotic machinery was altered at the expression level but not activated by maternal separation.

The adaptor protein FADD, netrin receptor and TNFAIP8, whose expression levels were affected by maternal separation (Additional file 5: Table S3), represent members of the extrinsic apoptotic pathway. The appearance of FADD in hippocampus of juvenile rats after maternal separation correlated with the simultaneous appearance of the netrin receptor in the same experimental group. In fact, FADD exhibited altered expression in all three brain regions of male rats. Whereas FADD and TNFAIP8 appeared in cortex, both these proteins disappeared from cerebellum of adult male rats previously subjected to maternal separation. TNFAIP8 disappeared from cerebellum of maternally separated juvenile as well as adult male and female rats suggesting that maternal separation increases susceptibility of the cerebellum to activation of caspase-8 and may promote the initiation of the extrinsic apoptotic pathway. Interestingly, the proteome profile of the extrinsic apoptotic pathway in the adult female brain was affected to a lesser extent by maternal separation. Because activation of the death receptor Fas and Fadd protein is followed by activation of caspase-8, we assessed activation of caspase-8. However, Western blot analysis indicated that the level of procaspase-8 (55 kDa) in brain tissue samples from animals affected by maternal separation was not changed. Furthermore, no detectable cleavage of procaspase-8 suggests that the extrinsic apoptotic pathway was not activated by maternal separation.

Several proteins interacting with p53 (tumor protein p53-binding protein 2, alpha-crystallin B chain) or associated with p53 activation (pyruvate dehydrogenase kinase isozyme 2), p53 expression (Mpv17 protein, insulin receptor substrate 2, C-reactive protein, activity-dependent neuroprotector homeobox protein), p53 posttranslational modification (Myb-binding protein 1A) or p53 cleavage (calpain-1 catalytic subunit) displayed altered expression in different brain regions after maternal separation (Tables 3, 4 and 5) indicating that p53 signaling is also modulated by this early postnatal intervention.

The third apoptotic pathway is associated with ER stress and activation of caspase-12. We did not observe any significant changes in the expression of procaspase-12 (60 kDa) after maternal separation. We also did not detect any cleavage fragments of procaspase-12. Because all three detected caspases representing three distinct apoptotic pathways were not activated, it seems that prolonged maternal separation did not induce apoptosis, which is consistent with the findings based on Western blot analysis.

Some previous studies were preoccupied with the consequences of maternal separation on apoptosis in the hippocampal area. The separation of young pups from their mother for 6 h daily during PND 1–14 [57, 79] or 3 h daily during PND 3–21 [62] induced apoptosis in the dentate gyrus of the hippocampus, while a 15-min maternal separation daily during the first two postnatal weeks (PND 1–13) increased the expression of antiapoptotic factors [36]. Maternal deprivation for 24 h at PND12 raised the level of Bax and Bcl-X (Bcl-2-like 1 protein) and increased cell death of neurons and glia in the cortex, hippocampus and cerebellum of infant rats [35]. Maternal separation for 6 h during the first two postnatal weeks promoted apoptosis of hippocampal neurons, increased mRNA and protein levels of caspase-3 and Bax, decreased both mRNA and protein levels of Bcl-2 and p-CREB. It was argued that the increase in apoptosis was mediated via the ERK signaling cascade and downregulation of p-CREB under these conditions [63]. Here we detected CREB1 protein only in cerebellum and maternal separation suppressed its expression in adult male and female rats (Additional file 5: Table S3) suggesting the increased susceptibility of the adult rat cerebellum to apoptotic stimuli.

Our results indicate that 3 h of maternal separation during the first three weeks after birth affects the proteome profiles of both the intrinsic and extrinsic apoptotic pathways, mainly in the brain of adult rats. If prolonged maternal separation triggered apoptotic processes during the first three postnatal weeks, the increased cell death would be just transitory. The rather minor alterations observed later in the levels of apoptotic and anti-apoptotic proteins could modulate the susceptibility of neural cells to apoptotic stimuli. Diverse proteomic profiles of different brain tissue samples from different groups of separated rats suggest that this susceptibility is age-, sex- and brain region-dependent.

Developmental cell death progresses in similar fashion in cortex and hippocampus and a little bit differently in cerebellum [19]. Here, we compared the expression levels of proteins involved in cell death and redox homeostasis in the three brain regions of control juvenile rats or juvenile rats subjected to maternal separation (Additional file 11: Table S6). Less than 1.5-fold difference between the groups was considered as a similarity. In control rats, 18.9% of proteins with similar expression levels were found in cortex and hippocampus but only 1.4% or 3.9% of proteins similarly expressed in cortex and cerebellum or in hippocampus and cerebellum, respectively. In rats subjected to maternal separation, 20.3% of proteins with similar expression levels were found in cortex and hippocampus, but only 1.4% or 1.4% of proteins similarly expressed in cortex and cerebellum or in hippocampus and cerebellum, respectively. Similar developmental profiles of the apoptotic process in cortex and hippocampus, in contrast to cerebellum, may be at least partially based on a relatively high similarity in the expression levels of proteins involved in the regulation of cell death and oxidative state in these two brain regions. This similarity was maintained also in juvenile rats subjected to maternal separation. It is worth noting that the similarity in the expression levels of a protein in two brain regions was found in some cases only in control juvenile rats or juvenile rats subjected to maternal separation; e.g., the level of Bax was similar in cortex and hippocampus of control juvenile rats but this similarity disappeared in juvenile rats subjected to maternal separation because the levels of Bax in cortex and hippocampus increased after maternal separation, approximating the levels of Bax in cerebellum. The levels of caspase-3 were similar in hippocampus and cerebellum of control rats but in cortex and hippocampus of maternally separated rats. The similarity in expression of the NMDA1 and NMDA2B subunits in cortex and hippocampus was preserved in both rat groups. On the other hand, a novel similarity appeared in expression of the NMDA2A subunit in cortex and hippocampus after maternal separation.

### Autophagy

Some proteins (beclin-1, insulin receptor substrate 2, autophagy related protein Atg5) associated with other type of cell death, autophagy, were found to be altered by maternal separation in the cortex and hippocampus of adult rats (Additional file 5: Table S3). Upon autophagy stimulation, beclin-1 is released from Bcl-2 protein and triggers the formation of ATG5-ATG12-ATG16 multimeric complex, which is crucial for the development of the autophagosome [20]. In our conditions maternal separation resulted in the simultaneous disappearance of Atg5 and beclin-1 from male hippocampus suggesting that the adult male hippocampus of rats previously subjected to maternal separation may lose two essential members of the autophagic machinery and the ability to undergo autophagic cell death. Beclin-1-induced autophagy is inversely correlated with apoptosis in adult neuronal stem cells [13]. The observed loss of beclin-1 may thus increase susceptibility of the female cortex and the adult hippocampus to apoptotic stimuli.

### The link between apoptosis and autophagic cell death

The calpain system is an important negative regulator of autophagy, which mediates the crosstalk between apoptosis and autophagy [13]. Two members of the calpain system, calpain-1 catalytic subunit and calpain 5, were found in cerebellum of male rats previously subjected to maternal separation. Calpains are able to cleave members of the Bcl-2 protein family, such as Bid, Bcl-2, Bcl-XL [17], Bax [80] and caspases-3, -8 and -12 [81]. Caspase-12 localized in the endoplasmic reticulum is cleaved by calpain in Ca^2+^-dependent manner when ER stress leads to the release of Ca^2+^ from intracellular stores into the cytosol. Calpain cleaves Bid into its active form tBid and induces apoptosis in a Bax-dependent manner [17]. It also cleaves Bax into a potent pro-apoptotic 18-kDa fragment [80]. Here, we assessed the cleavage of Bid, Bcl-XL, Bax, caspase-3, caspase-8 and caspase-12 by Western blotting. There were no detectable cleavage fragments of all these proteins, which suggests that the calpain system in the male cerebellum was altered by maternal separation at the level of expression but not activation. Uncontrolled calpain-mediated proteolysis of cellular proteins is prevented by specific inhibitor calpastatin, which may be cleaved by calpain or caspases. Here, we detected calpastatin in cortex of both juvenile and adult female rats affected by maternal separation (Table 3). Interestingly, the expression of this protein was inversely correlated with the disappearance of caspase-3 in cortex of maternally separated female rats. Caspase-3, Bax and beclin-1 were all undetectable in cortex of maternally separated female rats. Thus, maternal separation may suppress the apoptotic and autophagic processes in the female cortex and substitute them by other types of cell death.

### The role of glutamate receptors in cell death

The *N*-methyl-d-aspartate receptors (NMDARs) belong to a class of ionotropic glutamate receptors which play a significant role in mediating excitotoxic neuronal death or survival [82]. NMDARs are tetrameric complexes comprised of two essential Grin1 subunits and two Grin2 subunits or relatively rare GluN3 subunits [83]. Here we identified Grin1, Grin2A, Grin2B and Grin2C subunits. The essential Grin1 subunit was present in all brain regions and experimental groups and it became undetectable after maternal separation in the male cerebellum. The Grin2A and Grin2B subunits are the major Grin2 subunits expressed in the cortex and hippocampus [82]. Maternal separation affected the levels of both these subunits in cerebellum of juvenile and adult male rats (Additional file 5: Table S3). Whereas the Grin2A subunit was undetectable in cerebellum of control juvenile, male and female rats, maternal separation induced the expression of this subunit in juvenile and adult male but not female rats. The Grin2B subunit was present in control juvenile but not in adult rats of both sexes. Following maternal separation, this subunit disappeared from cerebellum of juvenile rats and appeared in this brain structure of adult male rats. The Grin2C subunit was detected only in cerebellum of juvenile, male and female rats affected by maternal separation (Table 5). Overall, prolonged maternal separation had a great impact on the expression of NMDAR subunits in the cerebellum. The NMDARs containing Grin2A and Grin2B promote neuronal survival and death, respectively [82]. The Grin2C subunit is specifically enriched in cerebellar granule cells and supports neuronal survival [63]. It is noteworthy that cerebellar neurons in control juvenile rats are apparently more prone to cell death while maternal separation promotes their survival. Prolonged maternal separation modulated pro-survival and death signaling via NMDARs in the male cerebellum and supported neuronal survival in the female cerebellum. Different models of maternal separation may cause differences in the expression of NMDARs and receptor signaling because the 24-h maternal deprivation on PND 9 reduced the mRNA levels of Grin2A and Grin2B subunits in the hippocampus and prefrontal cortex of adult rats [84]. In another study, no alterations in the mRNA levels of Grin2A and Grin2B were detected in the prefrontal cortex and hippocampus of adult mice subjected to 15-min or 3-h maternal separation for the first two weeks [85] suggesting that the impact of maternal separation on proteomic profiling of brain tissue may be mediated at the level of protein synthesis, folding, processing and degradation.

Yet another glutamate receptor altered by prolonged maternal separation was the metabotropic glutamate receptor 4 (mGluR4), which appeared in hippocampus of maternally separated juvenile and adult male rats and disappeared from prefrontal cortex of adult male and female rats previously subjected to maternal separation (Tables 3 and 4). Importantly, the decreased gene expression of mGluR (Grm4) was shown to be associated with neuronal apoptosis [86].

Glutamate may promote neural survival by acting through NMDARs and mGluRs [13]. On the other hand, glutamate is a well known trigger of early necrosis and delayed apoptosis in neurons [71]. It seems that prolonged maternal separation can modify the processes of survival and cell death induced by glutamate by different ways dependent on the brain region, either via altered expression of mGluR4 in the cortex and hippocampus or via altered expression of NMDAR subunits in the cerebellum.

### Non-apoptotic functions of molecular components mediating cell death

It seems that the apoptotic machinery is associated with local refinement of neural circuits without causing cell death. Local activation of caspases is involved in axonal degeneration and dendrite pruning [15]. Caspase-3 and Bax are essential for pruning and dysregulation of these proteins has been implicated in neurological disorders, such as schizophrenia and autism. Besides caspase-3 and Bax, the NMDA receptors are crucially involved in synaptic plasticity [87]. The caspase-3-dependent control of synaptic plasticity relies on Bax-mediated mitochondrial membrane permeabilization in order to promote NMDAR-dependent long term potentiation. The Bax-induced mitochondrial depolarization is mediated by NMDAR-induced calcium entry and calcineurin-dependent dephosphorylation of Bad [88]. We noted maternal separation-induced alterations in the levels of calcipressin-1 and calcipressin-2 in all three brain regions (Tables 3, 4 and 5). Both subtypes of calcipressin regulate calcineurin activity by direct binding and inhibition [89]. It seems that prolonged maternal separation may induce specific changes in protein expression in different brain areas that are age- and sex-dependent. Some of the differentially expressed proteins may participate in the modulation of synaptic plasticity and pruning. The GO enrichment analysis revealed a high enrichment of proteins engaged in the regulation of synaptic plasticity (Fig. 4c, Additional file 2: Table S1). Twelve proteins were detected in this protein class. The alterations of Creb1 and ionotropic glutamate receptors (Grid2, Grik2, Grin1, Grin2A and Grin2B) are shown in Tables 3, 4 and 5. The other seven proteins (neurabin-1, neuroligin-1, Shank1 protein, kalirin, PKCζ, metabotropic glutamate receptor 5 and protein S100-B) and changes in their expression induced by maternal separation are listed in Table 6. Two synaptic scaffolding proteins, neurabin-1 and Shank1, were decreased in hippocampus of adult male and female rats and increased in cerebellum of adult male rats. The Shank1 protein located at the post-synaptic density of glutamatergic synapses is essential for synaptic development and functions, and alterations in its expression lead to abnormal synaptic development causing a number of neuronal diseases [90]. The Shank1 protein was shown to interact with the glutamate receptor δ2 in cerebellar Purkinje cells [91]. In our study, both these proteins were upregulated in cerebellum of adult male rats subjected to maternal separation, suggesting potential changes in the modulation of cerebellar synaptic plasticity and synapse formation.

**Table 6:** A list of differentially expressed proteins involved in regulation of synaptic plasticity

Protein ID	Gene name	Protein name	Brain region	Y S/C	M S/C	F S/C	C M/F	S M/F
P97924; F1LZV1	Kalrn	Kalirin	Cerebellum	N	C	N	M	N
P31424 A0A0H2UHN1 A0A0H2UHW6	Grm5	Metabotropic glutamate receptor 5	Cerebellum	C	N	N	N	N
O35867	Pp1r9a	Neurabin-1	Hippocampus	-	↓ (0.50)	↓ (0.48)	–	–
Q62765	Nlgn1	Neuroligin-1	Cortex	↑ (2.10)	–	–	–	–
A0A0H2UHW8			Cerebellum	–	S	–	F	↓ (0.45)
P09217	Prkcz	Protein kinase C zeta type	Cortex	N	N	S	N	F
P04631	S100b	Protein S100-B	Hippocampus	–	–	↑ (2.19)	–	↓ (0.47)
			Cerebellum	–	–	–	–	↓ (0.25)
Q9WV48	Shank1	SH3 and multiple ankyrin repeat domains protein 1	Cerebellum	–	↑ (2.01)	–	–	–

*C* protein detected only in control samples, *S* protein detected only samples from maternally separated rats, *M* protein detected only in samples from adult male rats, *F* protein detected only in samples from adult female rats, *N* protein not detected in the samples; –, unchanged protein expression between the samples; ↓, protein down-regulated in samples from maternally separated rats; ↑, protein up-regulated in samples from maternally separated rats

### Limitations of the study

There are two major limitations in this study. The first limitation lies in using the combination of male and female brain tissues for juvenile analysis. It can be supposed that some differences in protein expression between juvenile males and females could appear at PND22. Unfortunately, this important aspect was not taken into account in the present work. Therefore, it is difficult to draw definite conclusions from the results obtained by analyzing brain tissue samples from juvenile rats of both sexes together. It is evident, however, that the brain proteomes of juvenile animals differed markedly from the proteomes of both adult male and female rats. In any case, it would be more appropriate to distinguish between the brain proteomes of juvenile male and female rats to uncover the potential differences in protein profiles between both sexes at a young age. The second limitation is that the mass spectrometry-based proteomics data were derived only from a single determination performed on one pooled sample of each experimental group and thus did not allow for appropriate statistical analysis and reliable assessment of significant alterations in rat brain proteome induced by maternal separation. Because there were large sets of animals (30 juvenile and 20 adult rats in each group), it would not be feasible to use every single sample for different analyses due to an insufficient amount of tissue. Besides that, mass spectrometric analysis of many individual samples would be highly demanding and extremely resource-intensive. These limitations might be overcome in future studies by pooling samples from several subsets of animals in each group. By using this strategy, biological variation would be at least partially preserved. It can be mentioned here that because of using a large number of animals in each group, the detected changes in protein expression were not random. Nevertheless, the current proteomic data do not provide characterization of biological variation but rather indicate trends in protein expression.

## Conclusion

Prolonged maternal separation may have a great impact on cell death-related protein expression profiling in different brain regions of both juvenile and adult rats. Many protein alterations were particularly detected in cerebellum from both age groups. The impact of maternal separation on cerebellum have so far been rarely studied. Therefore, greater attention should be paid to this brain region in future studies dealing with maternal separation. Because a 3-week regimen of maternal separation apparently did not elicit oxidative stress and cleavage of some caspases and other members of the apoptotic machinery, we assume that if apoptosis or autophagic cell death was triggered by this type of early postnatal stress in the course of the first three weeks, the activation of these processes would be only transitory and suppressed by adaptive responses. The changes observed in expression levels of proteins involved in cell death-related processes indicate that prolonged maternal separation could modulate responses to apoptotic and autophagic stimuli. Apoptosis has been monitored in rat cortex, hippocampus and cerebellum throughout the life cycle [19]. We suppose that the normal apoptotic activity at PND 21 and PND 90 is not affected by previous prolonged maternal separation. On the other hand, the distinct cell death proteomic profiles of control and maternally separated rats suggest that if cortical, hippocampal or cerebellar cells receive some apoptotic or autophagic stimuli the apoptotic or autophagic responses to these stimuli would be modulated rather differently. Our results support the notion that caspase-dependent and caspase-independent mechanisms of neuronal cell death are brain region- and age-dependent [14]. Because the proteome profiles obtained from cortical, hippocampal and cerebellar tissues differed between adult male and female rats it can be assumed that sex has a fundamental impact on the response to maternal separation. The differences observed between adult maternally separated rats and age-matched controls suggest that the consequences of maternal separation in the early postnatal period last into the adulthood. Our data characterizing the effect of maternal separation on the expression of cell death-related proteins in rat brain conform well to the match/mismatch hypothesis.

## Supplementary Information

Below is the link to the electronic supplementary material.

**Additional file 1: Fig. S1.** An overview of gene ontology (GO) enrichment analysis of biological processes.for up- and down-regulated proteins after maternal separation.

**Additional file 2: Table S1.** GO enrichment analysis of biological processes.

**Additional file 3: Fig. S2.** Functional enrichment analysis of the biological process GO terms. GO enrichment analysis was performed using ShinyGO v0.61 tool (*bioinformatics.sdstate.edu/go/*). For each experimental group, the cutoff of p-value (FDR) was set to 0.05. Hierarchical clustering trees complemented with p-values of the 50 most significantly enriched GO terms for biological processes in cortex, hippocampus and cerebellum of juvenile (A–C), male (D–F) and female (G–I) rats are shown. Blue dots of different sizes were generated automatically by the program and indicate the magnitude of p-value for each GO term (the lower p-value, the bigger dot). A. Hierarchical clustering tree summarizing the top 50 most significantly enriched GO terms that were identified in cortex of juvenile rats. The dataset analyzed consisted of 428 differentially expressed proteins with null q-value. B. Hierarchical clustering tree summarizing the top 50 most significantly enriched GO terms that were identified in hippocampus of juvenile rats. The dataset analyzed consisted of 313 differentially expressed proteins with null q-value. C. Hierarchical clustering tree summarizing the top 50 most significantly enriched GO terms that were identified in cerebellum of juvenile rats. The dataset analyzed consisted of 358 differentially expressed proteins with null q-value.D. Hierarchical clustering tree summarizing the top 50 most significantly enriched GO terms that were identified in cortex of male rats. The dataset analyzed consisted of 487 differentially expressed proteins with null q-value. E. Hierarchical clustering tree summarizing the top 50 most significantly enriched GO terms that were identified in hippocampus of male rats. The dataset analyzed consisted of 522 differentially expressed proteins with null q-value. F. Hierarchical clustering tree summarizing the top 50 most significantly enriched GO terms that were identified in cerebellum of male rats. The dataset analyzed consisted of 431 differentially expressed proteins with null q-value. G. Hierarchical clustering tree summarizing the top 50 most significantly enriched GO terms that were identified in cortex of female rats. The dataset analyzed consisted of 355 differentially expressed proteins with null q-value. H. Hierarchical clustering tree summarizing the top 50 most significantly enriched GO terms that were identified in hippocampus of female rats. The dataset analyzed consisted of 428 differentially expressed proteins with null q-value. I. Hierarchical clustering tree summarizing the top 50 most significantly enriched GO terms that were identified in cerebellum of female rats. The dataset analyzed consisted of 352 differentially expressed proteins with null q-value.

**Additional file 4: Table S2.** A survey of 586 proteins involved in regulating cell death, apoptosis and redox balance according to GO biological processes and antioxidant proteins according GO molecular function.

**Additional file 5: Table S3.** A list of 271 proteins excerpted from Table S2 whose levels were changed by prolonged maternal separation.

**Additional file 6: Table S4.** A list of proteins whose levels were different in control samples from adult male and female rats and these differences disappeared in rats subjected to maternal separation.

**Additional file 7: Table S5.** A list of proteins whose levels were equal in control samples from adult male and female rats and sex-dependent differences were induced by maternal separation.

**Additional file 8: Fig. S3.** Effect of prolonged maternal separation on the levels of glutathione (GSH), protein carbonyls, malondialdehyde (MDA) and lipid hydroperoxides (LOOH). All these markers were determined in the cerebral cortex, hippocampus and cerebellum of control (C) and maternally separated (S) rats. Samples of brain tissue were prepared from both juvenile (22) and adult male (90M) or female (90F) rats. Data represent means (± S.E.M.) of at least three independent measurements performed in triplicate. Maternal separation apparently did not significantly affect the levels of these markers in brain tissue.

**Additional file 9: Fig. S4.** Effect of prolonged maternal separation on activity of glutathione peroxidase (GPx). GPx activity was determined in the cerebral cortex, hippocampus and cerebellum of control (C) and maternally separated (S) rats. Samples of brain tissue were prepared from both juvenile (22) and adult male (90M) or female (90F) rats. Data represent means (± S.E.M.) of at least three independent measurements performed in triplicate. Maternal separation apparently did not significantly affect GPx activity in brain tissue.

**Additional file 10: Fig. S5.** Effect of prolonged maternal separation on the levels of selected proteins involved in apoptotic processes. All these markers were determined in the cerebral cortex (Cx), hippocampus (H) and cerebellum (Cb) of control (C) and maternally separated (S) rats. Samples of brain tissue were prepared from both juvenile (**A**) and adult male (**B**) or female (**C**) rats. The relative levels of Bak, Bax, Bcl-XL, Bid, caspase 3, 8 and 12 were assessed by Western blotting. Data represent means (± S.E.M.) of at least three independent experiments and were expressed as percent of the corresponding control.

**Additional file 11: Table S6.** A list of similarly expressed proteins in the prefrontal cortex, hippocampus and cerebellum of control and maternally separated juvenile rats.

## Data Availability

All the data generated or analyzed during this study is available.

## Abbreviations

Cb: Cerebellum
CREB: CAMP-response element binding protein
CRH: Corticotropin releasing hormone
Ctx: Cerebral cortex
DNPH: 2,4-Dinitrophenylhydrazine
DNTB: 5,5′-Dithiobis (2-nitrobenzoic acid
ECL: Enhanced chemiluminescence)
ERK: Extracellular signal-regulated kinase
ESL: Early-life stress
F: Female
FOX: Ferrous oxidation-xylenol orange
GPx: Glutathione peroxidase
GSH: Glutathione
GO: Gene onthology
H: Hippocampus
HCD: High-energy collisional dissociation
HPA: Hippocampus-pituitary-adrenal
HRP: Horseradish peroxidase
M: Male
MDA: Malondialdehyde
MOMP: Mitochondrial outer membrane permeabilization
NMDA: *N*-Methyl-d-aspartate
LFQ: Label-free quantification
LOOH: Lipid hydroperoxide
NADPH: Nicotinamide-adenine dinucleotide phosphate
nLC-MS2: Nanoscale liquid chromatography coupled to tandem mass spectrometry
PBS: Phosphate buffered saline
PKC: Protein kinase C
PND: Postnatal day
SDS-PAGE: Sodium dodecyl sulfate–polyacrylamide gel electrophoresis
TBA: 2-Thiobarbituric acid
TCA: Trichloroacetic acid
