# Supplementary material for: Proteome profiling of different rat brain regions reveals the modulatory effect of prolonged maternal separation on proteins involved in cell death-related processes
Source: Biol Res. 2021 Feb 8;54:4. doi: 10.1186/s40659-021-00327-5 (PMC7871601; doi:10.1186/s40659-021-00327-5)

**Additional file 1: Fig. S1.** An overview of gene ontology (GO) enrichment analysis of biological processes.for up- and down-regulated proteins after maternal separation


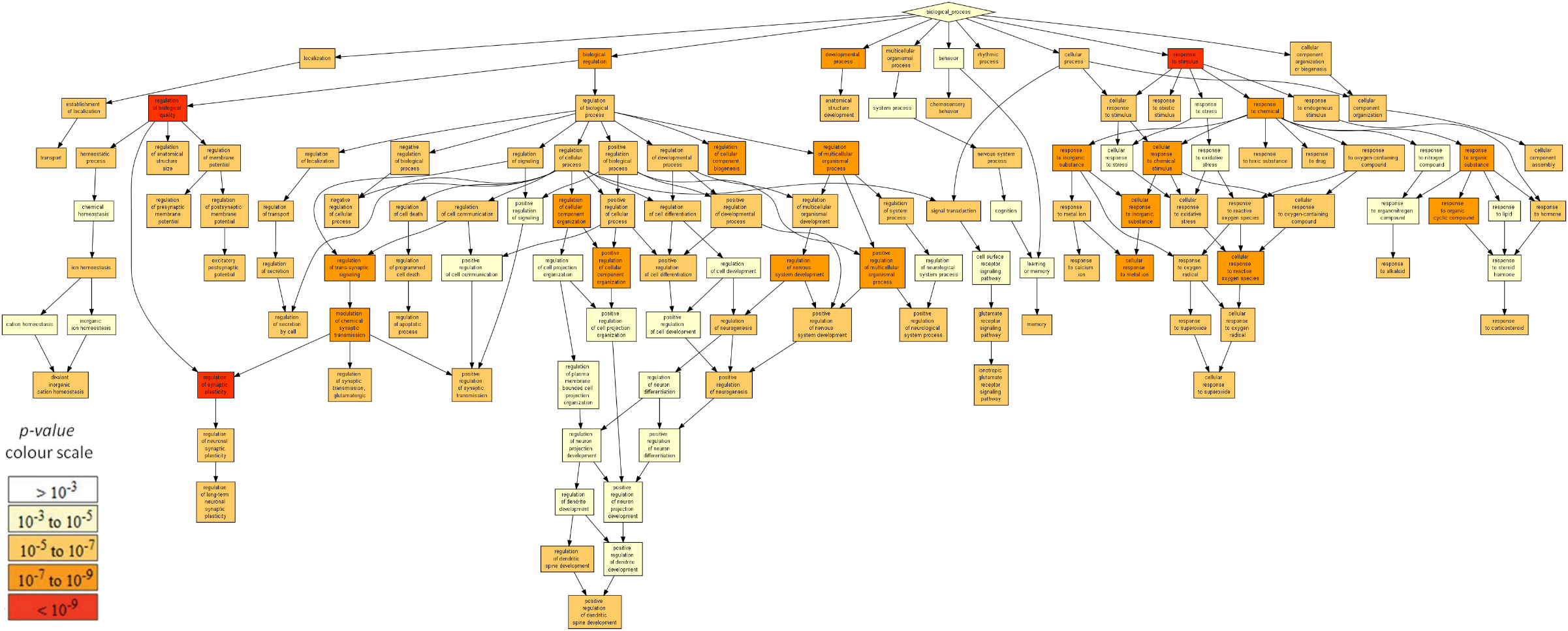

Supplement: Supplementary file 1 — Additional file 1: Fig. S1. An overview of gene ontology (GO) enrichment analysis of biological processes.for up- and down-regulated proteins after maternal separation. [file 40659_2021_327_MOESM1_ESM.docx]
