# Supplementary material for: Proteome profiling of different rat brain regions reveals the modulatory effect of prolonged maternal separation on proteins involved in cell death-related processes
Source: Biol Res. 2021 Feb 8;54:4. doi: 10.1186/s40659-021-00327-5 (PMC7871601; doi:10.1186/s40659-021-00327-5)

**Additional Fig. S2.** Functional enrichment analysis of the biological process GO terms. GO enrichment analysis was performed using ShinyGO v0.61 tool (*bioinformatics.sdstate.edu/go/*). For each experimental group, the cutoff of p-value (FDR) was set to 0.05. Hierarchical clustering trees complemented with p-values of the 50 most significantly enriched GO terms for biological processes in cortex, hippocampus and cerebellum of juvenile (A–C), male (D–F) and female (G–I) rats are shown. Blue dots of different sizes were generated automatically by the program and indicate the magnitude of p-value for each GO term (the lower p-value, the bigger dot).

**A.** Hierarchical clustering tree summarizing the top 50 most significantly enriched GO terms that were identified in **cortex of juvenile rats**. The dataset analyzed consisted of 428 differentially expressed proteins with null q-value.


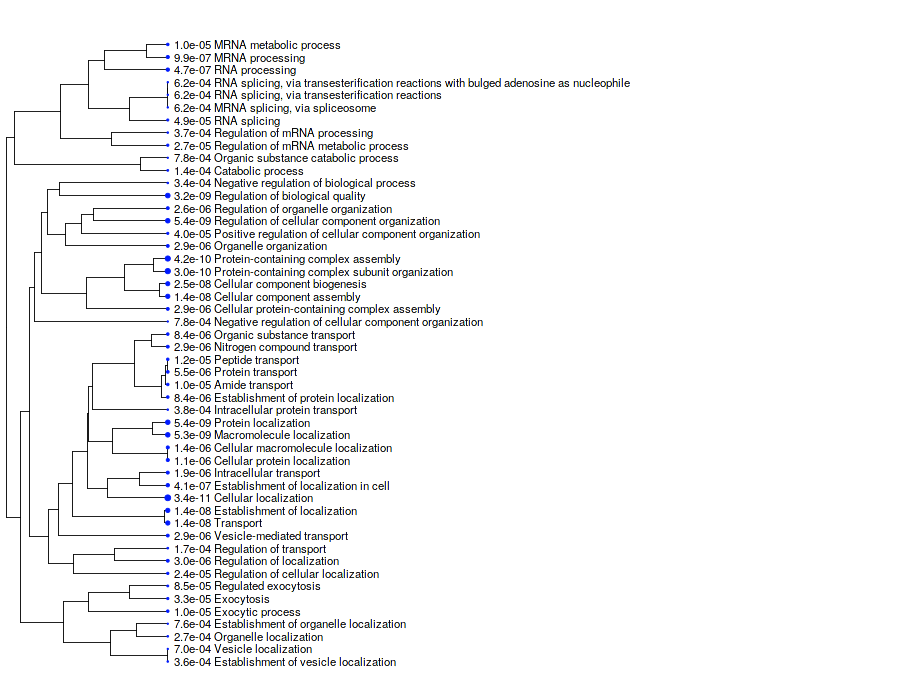


**B.** Hierarchical clustering tree summarizing the top 50 most significantly enriched GO terms that were identified in **hippocampus of juvenile rats**. The dataset analyzed consisted of 313 differentially expressed proteins with null q-value.


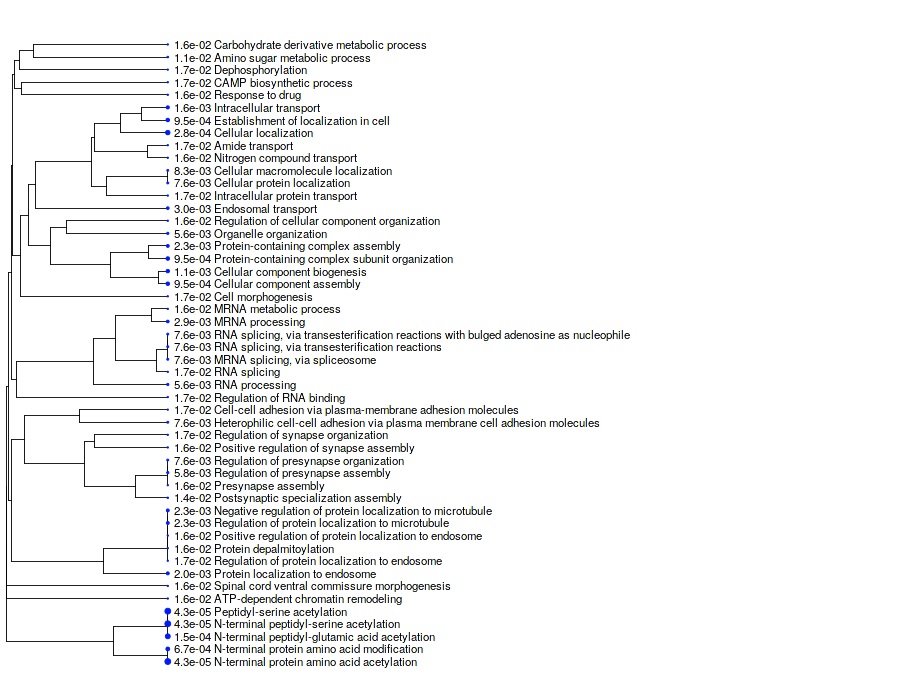


**C**. Hierarchical clustering tree summarizing the top 50 most significantly enriched GO terms that were identified in **cerebellum of juvenile rats**. The dataset analyzed consisted of 358 differentially expressed proteins with null q-value.


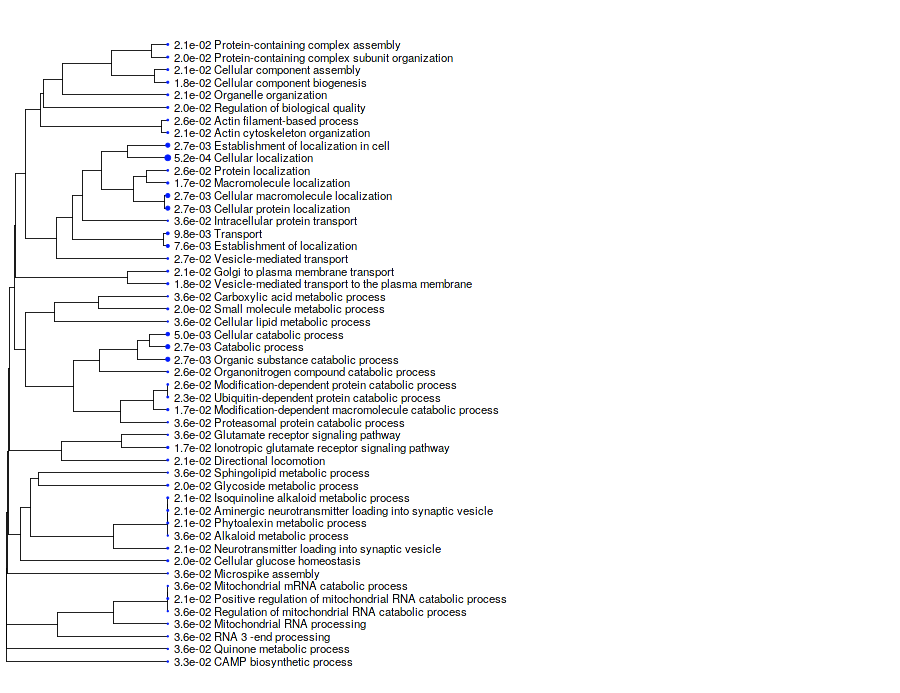


**D.** Hierarchical clustering tree summarizing the top 50 most significantly enriched GO terms that were identified in **cortex of male rats**. The dataset analyzed consisted of 487 differentially expressed proteins with null q-value.


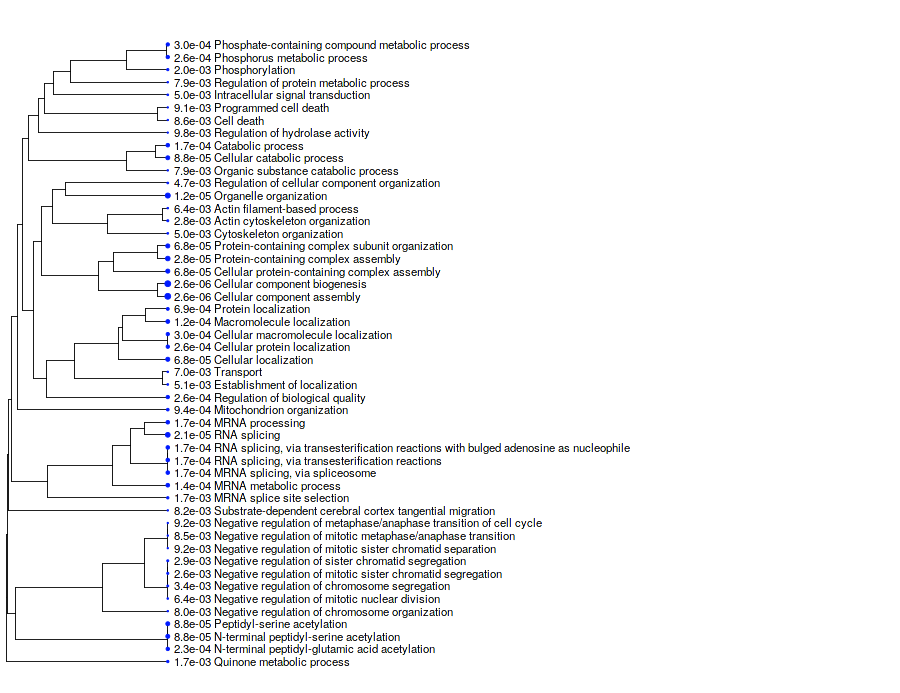


**E.** Hierarchical clustering tree summarizing the top 50 most significantly enriched GO terms that were identified in **hippocampus of male rats**. The dataset analyzed consisted of 522 differentially expressed proteins with null q-value.


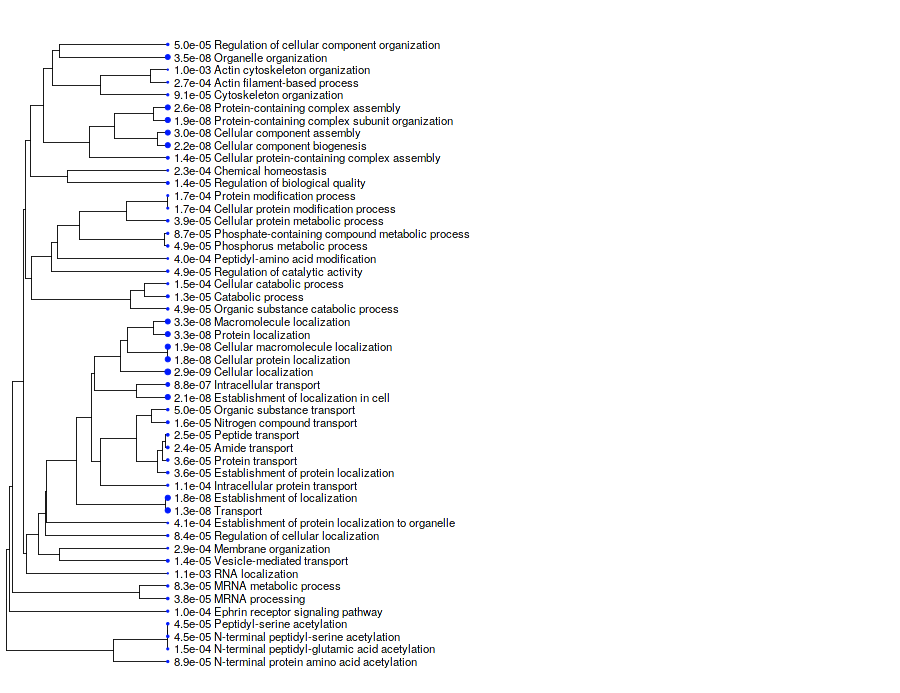


**F.** Hierarchical clustering tree summarizing the top 50 most significantly enriched GO terms that were identified in **cerebellum of male rats**. The dataset analyzed consisted of 431 differentially expressed proteins with null q-value.


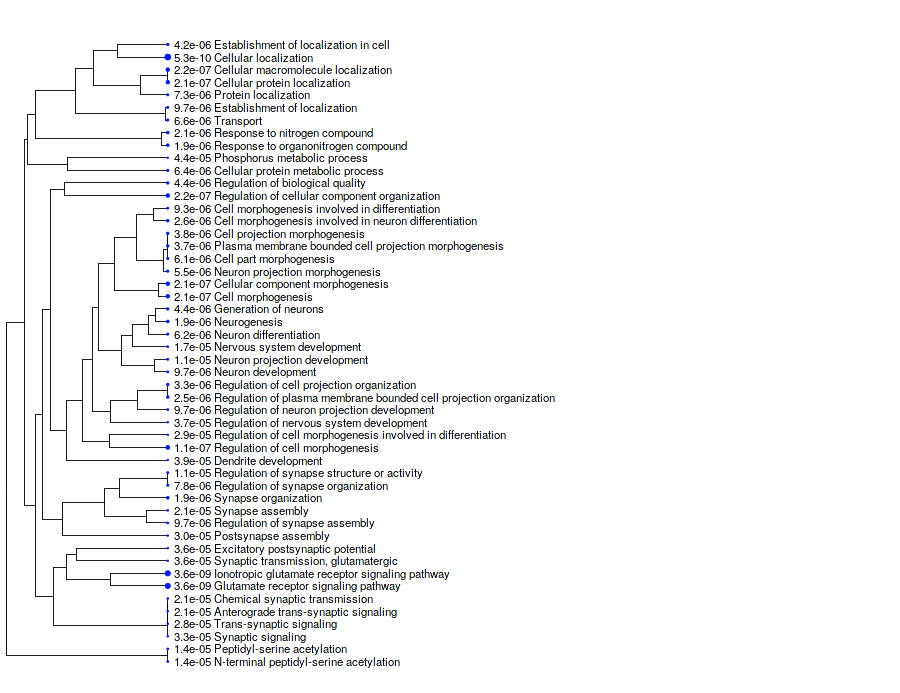


**G.** Hierarchical clustering tree summarizing the top 50 most significantly enriched GO terms that were identified in **cortex of female rats**. The dataset analyzed consisted of 355 differentially expressed proteins with null q-value.


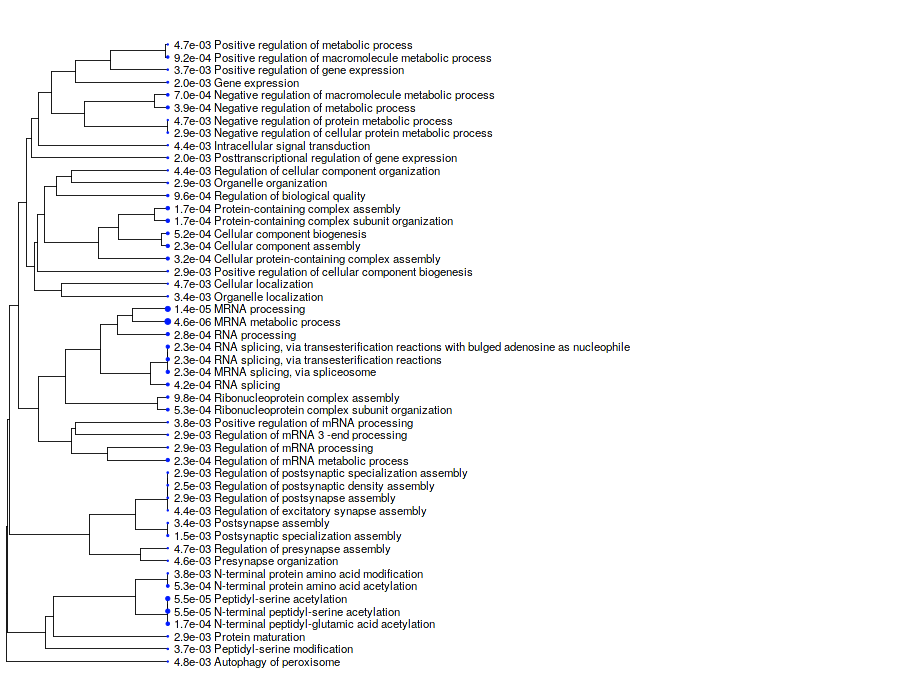


**H.** Hierarchical clustering tree summarizing the top 50 most significantly enriched GO terms that were identified in **hippocampus of female rats**. The dataset analyzed consisted of 428 differentially expressed proteins with null q-value.


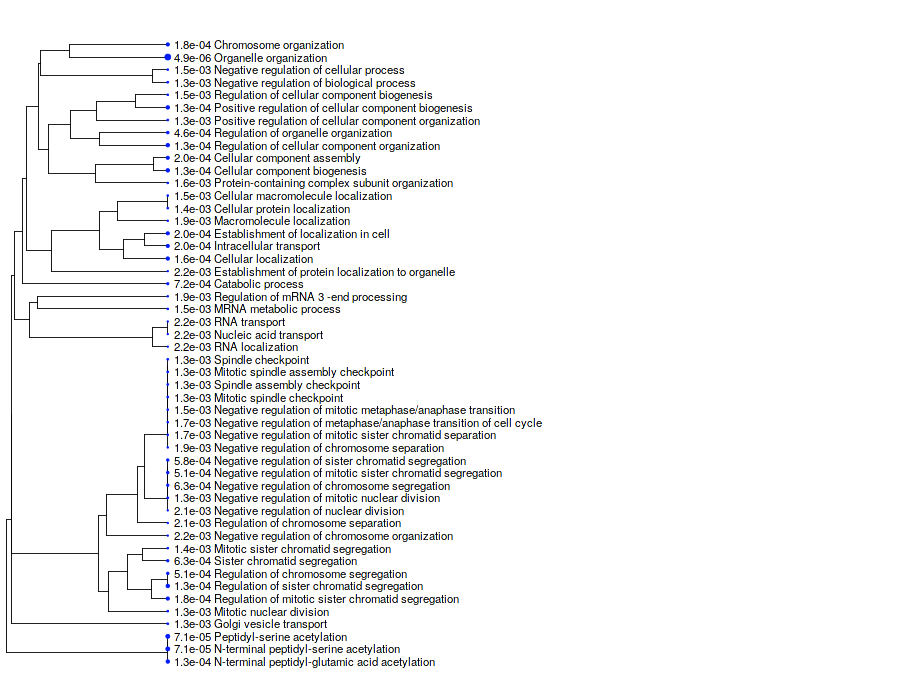


**I.** Hierarchical clustering tree summarizing the top 50 most significantly enriched GO terms that were identified in **cerebellum of female rats**. The dataset analyzed consisted of 352 differentially expressed proteins with null q-value.


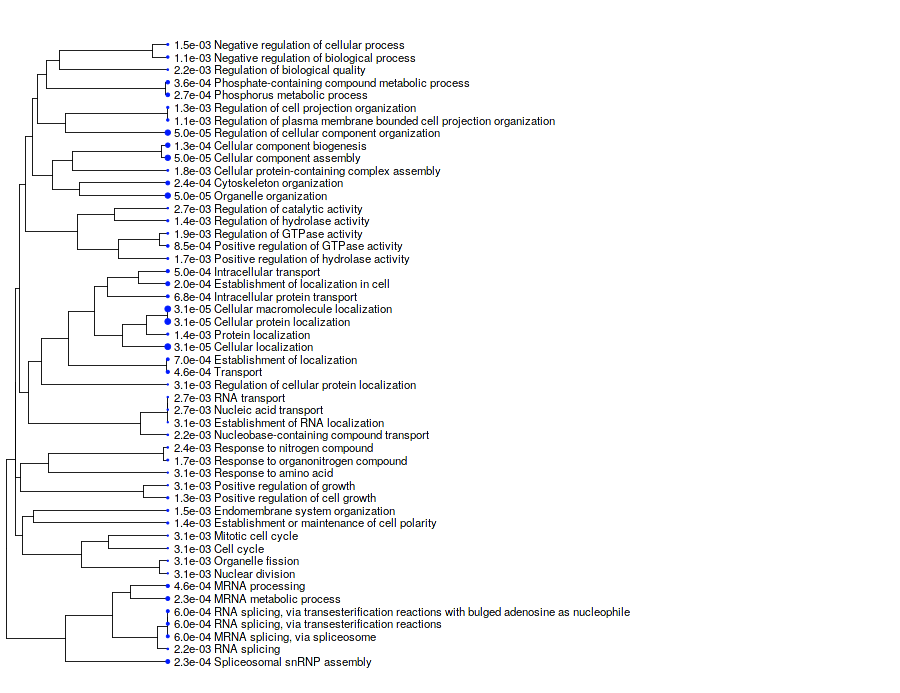

Supplement: Supplementary file 3 — Additional file 3: Fig. S2. Functional enrichment analysis of the biological process GO terms. GO enrichment analysis was performed using ShinyGO v0.61 tool (bioinformatics.sdstate.edu/go/). For each experimental group, the cutoff of p-value (FDR) was set to 0.05. Hierarchical clustering trees complemented with p-values of the 50 most significantly enriched GO terms for biological processes in cortex, hippocampus and cerebellum of juvenile (A–C), male (D–F) and female (G–I) rats are shown. Blue dots of different sizes were generated automatically by the program and indicate the magnitude of p-value for each GO term (the lower p-value, the bigger dot). A. Hierarchical clustering tree summarizing the top 50 most significantly enriched GO terms that were identified in cortex of juvenile rats. The dataset analyzed consisted of 428 differentially expressed proteins with null q-value. B. Hierarchical clustering tree summarizing the top 50 most significantly enriched GO terms that were identified in hippocampus of juvenile rats. The dataset analyzed consisted of 313 differentially expressed proteins with null q-value. C. Hierarchical clustering tree summarizing the top 50 most significantly enriched GO terms that were identified in cerebellum of juvenile rats. The dataset analyzed consisted of 358 differentially expressed proteins with null q-value.D. Hierarchical clustering tree summarizing the top 50 most significantly enriched GO terms that were identified in cortex of male rats. The dataset analyzed consisted of 487 differentially expressed proteins with null q-value. E. Hierarchical clustering tree summarizing the top 50 most significantly enriched GO terms that were identified in hippocampus of male rats. The dataset analyzed consisted of 522 differentially expressed proteins with null q-value. F. Hierarchical clustering tree summarizing the top 50 most significantly enriched GO terms that were identified in cerebellum of male rats. The dataset analyzed consisted of [file 40659_2021_327_MOESM3_ESM.docx]
