# Supplementary material for: Proteome profiling of different rat brain regions reveals the modulatory effect of prolonged maternal separation on proteins involved in cell death-related processes
Source: Biol Res. 2021 Feb 8;54:4. doi: 10.1186/s40659-021-00327-5 (PMC7871601; doi:10.1186/s40659-021-00327-5)
Supplement: Supplementary file 8 — Additional file 8: Fig. S3. Effect of prolonged maternal separation on the levels of glutathione (GSH), protein carbonyls, malondialdehyde (MDA) and lipid hydroperoxides (LOOH). All these markers were determined in the cerebral cortex, hippocampus and cerebellum of control (C) and maternally separated (S) rats. Samples of brain tissue were prepared from both juvenile (22) and adult male (90M) or female (90F) rats. Data represent means (± S.E.M.) of at least three independent measurements performed in triplicate. Maternal separation apparently did not significantly affect the levels of these markers in brain tissue. [file 40659_2021_327_MOESM8_ESM.docx]

**Additional Fig. S3.** Effect of prolonged maternal separation on the levels of glutathione (GSH), protein carbonyls, malondialdehyde (MDA) and lipid hydroperoxides (LOOH). All these markers were determined in the cerebral cortex, hippocampus and cerebellum of control (C) and maternally separated (S) rats. Samples of brain tissue were prepared from both juvenile (22) and adult male (90M) or female (90F) rats. Data represent means (± S.E.M.) of at least three independent measurements performed in triplicate. Maternal separation apparently did not significantly affect the levels of these markers in brain tissue.
