# Supplementary material for: Proteome profiling of different rat brain regions reveals the modulatory effect of prolonged maternal separation on proteins involved in cell death-related processes
Source: Biol Res. 2021 Feb 8;54:4. doi: 10.1186/s40659-021-00327-5 (PMC7871601; doi:10.1186/s40659-021-00327-5)
Supplement: Supplementary file 10 — Additional file 10: Fig. S5. Effect of prolonged maternal separation on the levels of selected proteins involved in apoptotic processes. All these markers were determined in the cerebral cortex (Cx), hippocampus (H) and cerebellum (Cb) of control (C) and maternally separated (S) rats. Samples of brain tissue were prepared from both juvenile (A) and adult male (B) or female (C) rats. The relative levels of Bak, Bax, Bcl-XL, Bid, caspase 3, 8 and 12 were assessed by Western blotting. Data represent means (± S.E.M.) of at least three independent experiments and were expressed as percent of the corresponding control. [file 40659_2021_327_MOESM10_ESM.docx]

**Additional Fig. S5.** Effect of prolonged maternal separation on the levels of selected proteins involved in apoptotic processes. All these markers were determined in the cerebral cortex (Cx), hippocampus (H) and cerebellum (Cb) of control (C) and maternally separated (S) rats. Samples of brain tissue were prepared from both juvenile (**A**) and adult male (**B**) or female (**C**) rats. The relative levels of Bak, Bax, Bcl-XL, Bid, caspase 3, 8 and 12 were assessed by Western blotting. Data represent means (± S.E.M.) of at least three independent experiments and were expressed as percent of the corresponding control.
